# A metagenomic prospective cohort study on gut microbiome composition and clinical infection in small bowel transplantation

**DOI:** 10.1080/19490976.2024.2323232

**Published:** 2024-03-04

**Authors:** Archana Madhav, Rachel Bousfield, Joana Pereira-Dias, Claire Cormie, Sally Forrest, Jacqueline Keane, Leanne Kermack, Ellen Higginson, Gordon Dougan, Harry Spiers, Dunecan Massey, Lisa Sharkey, Charlotte Rutter, Jeremy Woodward, Neil Russell, Irum Amin, Andrew Butler, Kayleigh Atkinson, Tom Dymond, Josefin Bartholdson Scott, Stephen Baker, Effrossyni Gkrania-Klotsas

**Affiliations:** a Cambridge Institute of Therapeutic Immunology and Infectious Disease, Department of Medicine, University of Cambridge, Cambridge, UK; b Department of Medicine / Department of Surgery, Cambridge University Hospitals NHS Foundation Trust, Cambridge, UK

**Keywords:** Small bowel transplantation, bacterial translocation, gut microbiome, sepsis, antibiotics, gut-origin sepsis, metagenomics, antimicrobial resistance

## Abstract

Two-thirds of small-bowel transplantation (SBT) recipients develop bacteremia, with the majority of infections occurring within 3 months post-transplant. Sepsis-related mortality occurs in 31% of patients and is commonly caused by bacteria of gut origin, which are thought to translocate across the implanted organ. Serial post-transplant surveillance endoscopies provide an opportunity to study whether the composition of the ileal and colonic microbiota can predict the emergence as well as the pathogen of subsequent clinical infections in the SBT patient population. Five participants serially underwent aspiration of ileal and colonic bowel effluents at transplantation and during follow-up endoscopy either until death or for up to 3 months post-SBT. We performed whole-metagenome sequencing (WMS) of 40 bowel effluent samples and compared the results with clinical infection episodes. Microbiome composition was concordant between participants and timepoint-matched ileal and colonic samples. Four out of five (4/5) participants had clinically significant infections thought to be of gut origin. Bacterial translocation from the gut was observed in 3/5 patients with bacterial infectious etiologies. In all three cases, the pathogens had demonstrably colonized the gut between 1–10 days prior to invasive clinical infection. Recipients with better outcomes received donor grafts with higher alpha diversity. There was an increase in the number of antimicrobial resistance genes associated with longer hospital stay for all participants. This metagenomic study provides preliminary evidence to support the pathogen translocation hypothesis of gut-origin sepsis in the SBT cohort. Ileal and colonic microbiome compositions were concordant; therefore, fecal metagenomic analysis could be a useful surveillance tool for impeding infection with specific gut-residing pathogens.

## Introduction

Small Bowel Transplantation (SBT) is a last-resort treatment for intestinal failure.^1^ SBTs are rarely performed; only 172 surgeries were performed globally in 2021.^2^ Twenty-seven of these were conducted across four specialist centers in the UK.^3^ In the UK, 88.2% and 72.6% of non-liver-containing SBT recipients are alive at one and three years respectively (70.7% and 52.0% for liver-containing grafts).^4^ Nine out of ten patients undergoing SBT develop clinically significant infections; nearly two-thirds of cases present with bacterial sepsis, commonly with enteric bacteria, making it the most frequent cause of morbidity and mortality in the post-transplant period.^5–7^

Gut-origin sepsis is a long-held hypothesis suggesting that damage to the intestinal barrier allows enteric pathogens or microbial products to translocate into the bloodstream, especially in patients undergoing abdominal surgery.^8–10^ In SBT patients, the use of empirical antimicrobials and immunosuppressive therapy to mitigate acute graft rejection inadvertently disrupts the gut microbiome, which enters a state of dysbiosis, in addition to damaging the structural integrity of the intestinal epithelium by the surgery itself.^7,11^ Furthermore, acute graft rejection is a common complication post-SBT, affecting 40% of recipients and accounting for 13% of patient deaths.^10,12^ Ischemia-reperfusion injury and damage to enterocytes that occurs as a result of acute graft rejection increase intestinal permeability, allowing for bacterial translocation, which can trigger a disseminated sepsis response.^13,14^ Graft-versus-host disease (GvHD) occurs in 9% of SBT recipients, 77% of whom die of the disease. Most GvHD-associated deaths are also caused by infection.^15^ Consequently, translocating antimicrobial-resistant (AMR) enteric pathogens are frequent etiologic agents of post-SBT clinical infections, currently posing a major barrier to improving patient outcomes. Prompt diagnosis and treatment of infections in SBT are crucial.

Gut microbiome alterations in SBT patients have previously been recognized as potential predictive markers of infection, sepsis or graft rejection.^16^ Reduced gut microbiome diversity has also been associated with adverse patient outcomes in other solid and liquid organ transplants; lower microbiome diversity in the post-operative period is associated with graft rejection and bacteremia after liver and kidney transplantation.^17,18^ In allogeneic stem cell transplantation, domination by *Enterococcus* and Proteobacteria in the gut is associated with an increased risk of developing Enterococcal or Gram-negative bacteremia.^19^ Since infection and rejection in SBT can present with similar clinical signs and symptoms, endoscopic biopsies are necessary to distinguish between them.^20^ Routine surveillance endoscopies, accessed through the stoma created during surgery, offer opportunities to monitor the recovery of the surgically altered bowel and sample the gut microbiome.^11^

The aim of this study was to perform whole-metagenome sequencing (WMS) to assess the gut microbiomes of five SBT recipients during the post-transplant period. The primary objective was to characterize the temporal shifts in microbiota composition and bacterial diversity with respect to clinical infections and determine whether the infective pathogen can be linked back to the gut microbiome at species or sequence-type (ST) level. We hypothesized that gut colonization precedes invasive infections by the dominant species. Secondary objectives included reporting the changes in 1) AMR carriage and 2) genes encoding functional metabolic pathways in gut bacteria after SBT.

## Methods

### Experimental design

This prospective cohort study enrolled and followed up with five adult participants undergoing Small Bowel Transplantation between August 2021 and December 2022. This single-center study involved the Multi-Visceral Transplant, Gastrointestinal and Infectious Diseases Departments at Cambridge University Hospital NHS Foundation Trust, UK. This study was approved by the Wales Research Ethics Committee (IRAS Project ID 213,655). Patients and the public were not involved in the design, conduct, reporting, or dissemination plans of the research.

### Participant eligibility criteria and consent

Eligible study participants were aged over 17 years, male or female, under the care of the Multi-Visceral Transplant team, and were able to give informed consent. The participants received written information, telephone contact with the study team, and completed an electronic consent form.

### Donor organ retrieval

The multi-visceral organ retrieval was performed after confirmation of brain death of donors. In each case, an 80 L cold box was used, which contained multiple 1 L Belzer UW® cold storage transport solution, Soltran bags and Hartmann’s fluid, packed with crushed ice. Heparin and antimicrobials were given during the retrieval operation and the organs were perfused with Belzer solution via the aorta and portal vein. All donors received pre-operative gastrointestinal decontamination (nasogastric antibiotic ciprofloxacin and antifungal fluconazole) and intra-operative intravenous antibiotic meropenem, vancomycin and antifungal amphotericin B liposomal.

Organs were further perfused with room temperature and then cold Hartmann’s infusions until cold and ready to be removed. They were placed in cold ice slush dishes and then packed for transport. The organ block was packed in 3 sterile bags with cold preservation solution, cold saline, and cold fluid respectively. This was placed in the 80 L cold box with ice, sealed with secure tape and labeled.

### Clinical data, sample collection and anonymisation

Clinical data on the recipients were collected from electronic medical records and included demographics, antimicrobial and immunosuppressant use, and clinical outcomes (infection or rejection episodes and survival). Data were collected on the day of transplantation and at post-transplant endoscopy appointments. The participants were followed up for 1 year to collect infection and survival data. Any organisms that were identified from clinical samples and reported as significant by a clinical microbiologist were included in the analyses.

Fecal effluent samples were collected from study participants on the date of transplantation (day 0) and at follow-up endoscopies. Samples collected at the time of transplantation included 10 mL of fecal effluent from the recipient’s discarded colon, 10 mL from the recipient’s discarded ileum and 10 mL of donor effluent sample after implantation from the new ileum (which came from the donor and was placed into the recipient). These were transferred on ice from the operating theater to laboratory fridges and processed within 36 hours.

Participants were assigned a unique number.^1–5^ Samples were assigned a unique code comprising the participant’s unique number, followed by the visit or endoscopy number (V1/E1-E5), and the site from which the sample was taken (ileal-I, colonic-C, donor-D), as shown in Supplementary Data, Table 1.

**Table 1.** Organisms cultured from clinical samples by participant.

Participant	Cultured organism causing infection	Sample type	Days post transplantation	Presumed organism origin
1	*Candida glabrata*	Abdominal drain fluid	22	Gut
2	Vancomycin resistant *Enterococcus faecium* Vancomycin resistant *Enterococcus faecium* Vancomycin resistant *Enterococcus faecium* *Staphylococcus epidermidis* *Staphylococcus epidermidis* *Staphylococcus epidermidis* Vancomycin resistant *Enterococcus faecium* Vancomycin resistant *Enterococcus faecium* Vancomycin resistant Enterococcus faecium*	Urine Blood Blood Blood Blood Blood Blood Line culture Blood	41 81 100 133 134 137 148 149 151	Gut Gut Gut Skin Skin Skin Gut Gut Gut
3	*Aspergillus fumigatus* Vancomycin resistant Enterococcus faecium* Pseudomonas spp. *Aspergillus fumigatus*	Sputum Abdominal body fluid Urine Bone biopsy	4 12 67 182	Lungs Gut Unclear Lungs
4	Vancomycin sensitive *Enterococcus faecium* Vancomycin resistant *Enterococcus faecium* *Klebsiella michiganensis* Klebsiella michiganensis* Vancomycin resistant *Enterococcus faecium*	Abdominal drain fluid Abdominal drain fluid Abdominal drain fluid Peritoneal fluid (surgical sample) Donor artery/vein (surgical sample)	7 13 13 15 15	Gut Gut Gut Gut Gut
5	None			

*Bacterial isolates that underwent whole-genome sequencing.

### Sampling considerations

It was impossible to enroll and consent donors into this study (prior to their unexpected deaths) and sample their organs (or collect any personal data) for this research study due to ethical considerations. There is no sampling of donor gut microbiomes performed in the UK health system routinely. However, immediately after surgical implantation, when the donor organ was perfused from the recipient’s blood supply, the donor’s bowel and effluent belonged to the recipient and therefore could be sampled for purposes of this research study. Sampling of the ‘donor’ fecal effluent was performed at this point. Effluent sampling was chosen over gut tissue sampling as it is considered less invasive, is known to contain abundant bacterial DNA and the authors hypothesized that it may translate to bedside stoma-site sampling in the future.

### Effluent sample processing

Neat effluent sample (500 µl) was added at a ratio of 1:1 with 500 µl of in-house-produced lysis buffer (4 M guanidine thiocyanate (Sigma-Aldrich®) in 25 mM Tris-HCl, 0.5% β-mercaptoethanol (Sigma-Aldrich®) into a Lysing Matrix E bead tube (Mp Biomedicals™, code 116,914,050.) This underwent bead beating using the FastPrep™ machine (Mp Biomedicals™) for 40 seconds at speed 6,000 rpm and then incubated at room temperature for 10 minutes.

Ethanol (100% (v/v), 1 mL) was added to the bead tube to achieve an ethanol concentration of 50% (v/v) and the tube was incubated at ambient temperature for a further 10 minutes with gentle agitation. A further 860 µl of lysis buffer was added to the tube to bring the final ethanol concentration to 35% (v/v) for optimal column binding. Then 700 µl of this mix was pipetted into a new spin column tube (NBS Biologicals Ltd, code SD5008). The spin columns were placed in the microfuge rotor and spun at 15,000 rpm for 1 minute. The flow-through was discarded.

The column was washed by adding 500 µl of Wash Buffer 1 (1 M guanidine thiocyanate in 25 mM Tris-HCl, with 10% ethanol) and spun for 30 seconds at 15,000 rpm, and the eluate was discarded. The column was washed again, using 500 µl of Wash Buffer 2 (25 mM Tris-HCl buffer, with 70% ethanol) and spun for 30 seconds at 15,000 rpm and the eluate was discarded. The column was washed once more, with 500 µl of Wash Buffer 2 and spun for 2 minutes at 15,000 rpm to remove all the wash solution. A summary of buffer compositions can be found in Supplementary Data.

After ensuring there was no liquid remaining in the tip of the spin column, the spin column was transferred to a clean RNAse free tube (ThermoFisher Scientific Inc., code AM12425.) The nucleic acids were eluted by adding 100 µl of nuclease free water to the column and spun at 15,000 rpm for 1 minute. The spin column was discarded, and the nucleic acid was stored at −70°C.

### Sequencing of bowel effluent samples

DNA concentrations were quantified on a Qubit™ 2.0 Fluorometer, and 50 µl of DNA from each sample was transferred into barcoded snap-cap microcentrifuge tubes and shipped to Eurofins Genomics for standard library preparation and sequencing. DNA samples were sequenced on an Illumina NovaSeq 6000 S4 PE150 instrument, with a minimum of 10 million paired-end reads requested for metagenomic sequencing of bowel effluent samples.

### Sequencing of selected bacterial isolates

Three recovered clinical bacterial isolates were taken from freezer and passaged twice, on Columbia blood agar (Merck), incubated at 37°C overnight, in aerobic conditions. *Enterococcus faecium* was grown for 48 hours prior to extraction. *Klebsiella michiganensis* was grown for 24 hours prior to extraction. Identification was confirmed using the MALDI-TOF Biotyper® (Bruker) platform. The QIAamp® DNA Mini Kit (Qiagen®, code 51,304) was used to extract the bacterial DNA as per the manufacturer’s protocol.

DNA concentrations were quantified on a Qubit™ 2.0 Fluorometer and 100 µl of DNA from each sample was transferred into barcoded snap-cap microcentrifuge tubes and shipped to Eurofins Genomics for standard library preparation and sequencing. Isolate DNA samples underwent whole-genome sequencing (WGS) on an Illumina NovaSeq 6000 S4 PE150 instrument, with a minimum of five million paired-end reads requested for each bacterial isolate.

### Bioinformatics analyses

The raw metagenomic sequencing reads were assessed for quality using FastQC v0.11.9.^21^ After checking QC statistics, kneaddata v0.10.0^22^ along with the FastQC outputs were used to remove adapters, primer sequences and host reads using the human GCh38 reference genome. Taxonomic assignment on the QC-checked reads was performed using Kraken2 v2.1.2^23^ using a custom-built database containing archaeal, bacterial, human, fungal, protozoan and viral reference genomes. The “extract_kraken_reads.py” script from the KrakenTools v1.2 suite was used to remove classified human host reads and Kraken2 was run again on the filtered reads for microbial assignment. Bayesian re-estimations at phylum and species levels were performed using Bracken v2.7.0.^24^ Following assignments, archaeal, viral, and protozoan phyla were disregarded due incomplete draft reference genomes and because our extraction protocol was not optimized to capture organisms belonging to these kingdoms.

Raw metagenomic reads were assembled into contigs using metaSPAdes v3.13.0,^25^ followed by AMR gene detection using ABRIcate v1.0.1^26^ with the Comprehensive Antimicrobial Resistance Database (CARD)^27^ as reference. The ABRIcate pipeline identified 916 AMR genes in the sample collection. We filtered these to only consider genes detected with > 95% identity to the CARD reference gene, producing a subset of 746 AMR genes. In addition, AMR genes conferring resistance to ≥ 3 drug classes were reclassified as ‘multidrug’. An unpaired t-test was used to show statistically significant differences (*p* < 0.05) in the total number of AMR genes between samples pooled by timepoint for the first three timepoints, i.e., day of transplant, first, and second follow-up endoscopies. Functional profiling was carried out to detect genes corresponding to metabolic pathways by running HUMANN3 v3.5 against the Uniref90 database and normalized to reads per kilobase mapped (RPKM) values.^22^ MaAsLin2 v1.12.0 was used to extract significant associations (if any) between participants and metabolic pathway gene abundances.^22^ Participant 5 was selected as the reference comparator for gut bacterial functional pathway gene abundance calculations. Participant 5 had the best outcome post-transplant, defined as no post-operative sepsis or rejection episodes, survival at 1 year, and short inpatient hospital stay.

Downstream analyses were performed in RStudio v4.0.4. Kraken2 reports were visualized on the Pavian R package v1.2.0.^28^ Data cleaning and transformation were carried out using the dplyr v 1.0.8,^29^ stringr v1.4.0, tidyr v1.2.0 and tidyverse v1.3.2 packages.^30^ Microbial alpha and beta diversities were computed using vegan v2.5–7.^31^ Bracken results were combined into a biom table format and rarefied to randomly subsample 20,477 operational taxonomic units (OTUs) from each sample without replacement. PCA was performed using the stats v4.0.4^32^ and ggbiplot v0.55^33^ packages to assess total variation in the data.

There was wide variability in the number of raw metagenomic reads per sample among the effluent sample collection (Range: 15956–13071245) and the number of operational taxonomic units (OTUs) detected (Range 931–11811807). We rarefied the collection to randomly subsample at an arbitrary threshold of 20,477 OTUs. Three samples (1V1I, 3E3C and 3E3I) had fewer OTUs than the designated threshold, for which we used all available data on assigned OTUs for individual sample composition analyses, but these samples were excluded from diversity, AMR and metabolomic analyses.

Raw WGS reads from the three bacterial isolates were processed using Nextflow v21.10.6^34^ and the GHRU *de novo* assembly pipeline v2.1.2.^35^ The inStrain v1.8.0 pipeline^36^ was used to link isolates from clinical infection episodes back to patients’ gut microbiomes. FASTA files from the three WGS isolates were indexed using bowtie2 v2.4.5.^37^ Raw read files from the bowel effluent samples containing the highest relative abundance of the corresponding organisms were selected according to the patients and timepoints these infections manifested in. Bowtie2 was then used to map the raw read files against the indexed isolate sequences. The inStrain profile and inStrain compare functions were used to determine average nucleotide identity (ANI) among the regions from the effluent reads which mapped to the indexed isolate genomes (Supplementary data, table s2). The ‘coverage overlap’ column denotes the percentage of bases in the indexed genome which mapped to the effluent read samples. The ‘percent compared’ column depicts the fraction of the reads sequenced at ≥5× coverage in both effluent samples that were being compared to the indexed genome.^36^ ANI and relatedness between the two *Enterococcus faecium* isolates were tested using FastANI v1.33^38^ and MASH v2.3.^39^ Finally, the two *Enterococcus faecium* isolates were sequence-typed using MLST v2.23.0^40^ on the command line.

### Bias and sample size

We reduced bias in our cohort study by utilizing remote, real-time enrollment to all those listed for transplantation at our center, and consequently, none declined participation. We reduced the loss to follow-up to zero by sampling along with routine clinical procedures performed in the hospital setting. A sample size of five was chosen to ensure that time and financial constraints enabled completion over 18 months, recruiting approximately one participant per month and allowing year-long follow-up.

## Results

### Participant clinical details

Multiple sampling of the five participants over time allowed the collection of 40 bowel effluent samples in total: 5 from post-implantation donor grafts on the date of transplant, 17 from recipient ileums, and 18 from recipient colons. At the time of transplantation, all participants were receiving parenteral nutrition, were ex-smokers or nonsmokers, and had alcohol intake within the recommended limits. None of the participants had serological evidence of previous infection with hepatitis B or C and none had active COVID-19 infection, which was excluded by concurrent PCR testing.

### Total microbiota composition

**Figure 1.**
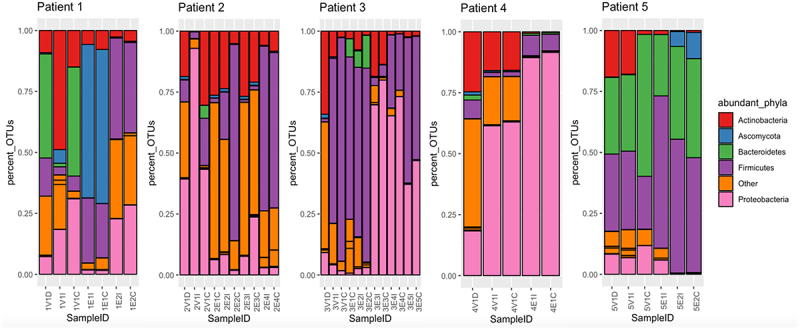
Stacked bar charts representing the relative abundances (% of OTUs in sample) of five most prominent phyla by sampling timepoint.

65 unique phyla were detected in the 40 samples. The top nine phyla accounted for over 96% of all operational taxonomic units (OTUs) detected in the collection. Of these, we selected the five most abundant bacterial and/or fungal phyla and assessed their relative abundances longitudinally. Figure 1 shows the most abundant phyla in each sample. The most abundant phyla were Proteobacteria (50.4%), Firmicutes (30.4%), Bacteroidetes (11.2%), Ascomycota (2.3%) and Actinobacteria (1.7%).

Samples are arranged longitudinally by participant, beginning with the donor sample. Samples were assigned a unique alphanumeric identification comprising the participant’s unique number (1,2,3,4,5), followed by the transplantation visit (V1) or endoscopy number (E1-E6) and the sample type (ileal-I, colonic-C, donor-D.)

### Gut microbiota composition and clinical outcomes

In our study, the gut microbiota composition was predictive of subsequent episodes of clinical infection. Enteric pathogens showed high relative abundances in the gut between 1–10 days prior to causing invasive clinical infection. Four out of the five (4/5) participants had clinically significant organisms identified (Table 1). Three out of six (3/6) of these organisms were thought to be of gut origin: *Candida glabrata*, *Enterococcus* spp., and *Klebsiella michiganensis*. *Aspergillus fumigatus* in participant 3 was suspected to have originated from the lungs (isolated in sputum and later causing an episode of discitis), and *Staphylococcus epidermidis* in participant 2 was thought to have originated from the skin (later causing a line infection). The origin of the pseudomonal urinary infection in participant 3 was less clear and could include environmental (water) sources. The high Firmicutes abundance was often due to the dominance of *Enterococcus faecium*.

**Figure 2.**
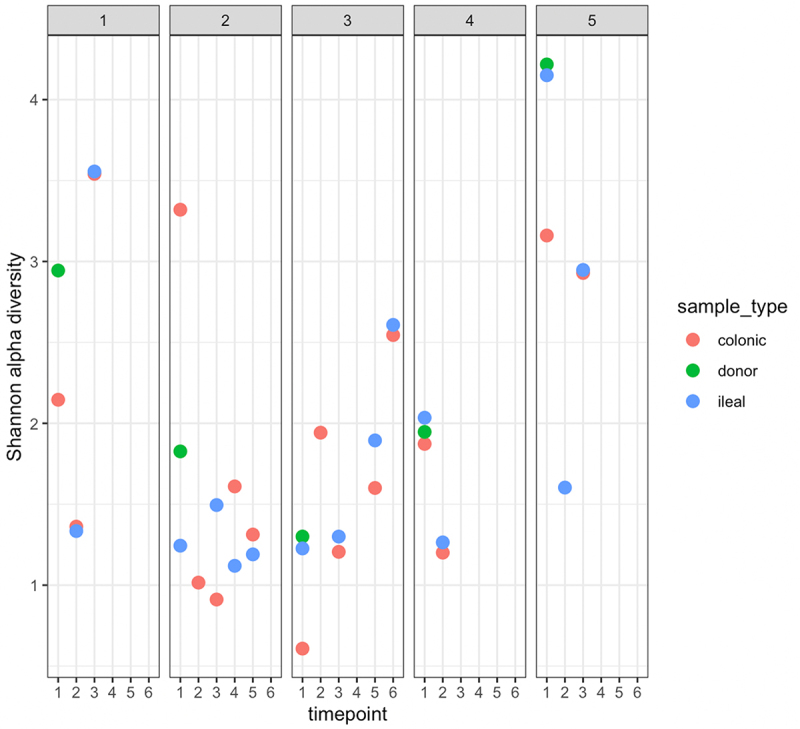
Shannon α-diversity index, at species level, of participant samples.

In our cohort, we noted that higher donor alpha diversities appeared to be associated with better recipient outcomes (Participants 1 and 5). Figure 2 shows the Shannon alpha diversity index at the species level for all available participant samples. Participants whose post-transplant ileal and colonic samples had Shannon alpha diversities increase to over 2.5 at their final endoscopy, i.e., participants 1,3 and 5, survived. Pavian plots containing the metagenomic compositions for each sample are shown in Supplementary Data, Figure 1.

Samples are arranged longitudinally by participant. Timepoint 1 corresponds to the date of transplant and all subsequent timepoints show α-diversity at follow-up endoscopies.

#### Participant 1

Effluent samples were collected at transplant (day 0) and at the first and second endoscopies (days 13 and 34, respectively). Over 40% of the donor sample comprised the Bacteroidetes phylum (42.65% of all assigned OTUs) – the highest composition of Bacteroidetes among all five donors in our cohort. The donor sample also had the second-highest alpha diversity among all donor samples (Shannon = 2.96). At the first follow-up endoscopy, samples from participant 1 showed an increase in the relative abundance of the phylum Ascomycota (dominated by *Candida glabrata*) in both the ileal (62.95%) and colonic (63.19%) samples. Ten days after the first endoscopy, the patient was diagnosed with a *Candida glabrata* intra-abdominal infection, requiring surgical drainage and antifungals. They achieved good recovery and alpha diversity of samples obtained at the second endoscopy had increased to 3.55. Participant 1 was discharged 53 days after their transplant.

#### Participant 2

Effluent samples were collected at transplant (day 0) and during four follow-up endoscopies (days 20, 40, 60, and 81). Samples collected on day 40 showed abundant *Enterococcus faecium* in the gut. The next day, vancomycin-resistant *Enterococcus faecium* (VRE) was detected in the urine. The participant was treated with tigecycline, and subsequent endoscopy (day 60) showed a reduction in the taxon in response to the treatment administered. On further endoscopy (day 81), both ileal and colonic samples showed a substantial relapse of VRE, and they developed *Enterococcus faecium* bacteremia post-procedure on the same day. The alpha diversity of both the ileal and colonic samples at this time point was below 1.5. They had two additional VRE bloodstream infections and sepsis episodes (days 148 and 151) possibly contributing to their death 164 days post-transplant.

#### Participant 3

Effluent samples were collected at transplant (day 0) and at five follow-up endoscopies (days 8, 15, 43, 84, and 99). At the time of transplantation, the recipient’s samples showed high abundances of *Enterococcus faecium* (77.04%–80.12%), and this taxon remained dominant at the first (50.59%) and second endoscopies (66.62%–71.02%). Three days after the first endoscopy, VRE was cultured from the body fluid of participant 3 (Table 1). *Aspergillus fumigatus* was detected in the sputum (on day 4 post-transplantation) and bone biopsy (on day 182) but was not observed in the gut microbiome. Notably, the donor sample for participant 3 had the lowest alpha diversity at the species level among all donor samples tested (Shannon index = 1.31), as shown in Figure 2. Participant 3 was discharged 144 days post-transplantation and was alive at 12 months follow up, despite a readmission at 7 months with fungal (Aspergillus fumigatus) discitis, requiring voriconazole, laminectomy and decompression.

#### Participant 4

Effluent samples were collected at transplant (day 0) and one follow-up endoscopy (day 10) prior to death on day 18. The donor sample for participant 4 had a Shannon alpha diversity under 2.0, and the recipient samples collected on day 0 showed similar alpha diversity indexes (1.86–2.04). Endoscopy samples had notable relative abundances of *Enterococcus faecium* (5.66%) and the genus *Klebsiella* (71.5% − 73.0%) which were classified by Kraken2 as *Klebsiella michiganensis*. *Klebsiella michiganensis* and VRE were isolated from the peritoneal fluid and blood clots on day 15, at the time of surgery, prior to death (Table 1). Therefore, both organisms that contributed to death were abundant in the gut prior to participant 4’s deterioration.

#### Participant 5

Participant 5 had the best outcomes: no infection/rejection episodes, survival and a short inpatient hospital stay. Samples were collected on the day of transplantation (day 0) and at the first and second follow-up endoscopies (day 13, 21). The donor sample for participant 5 had the highest alpha diversity index (Shannon = 4.20), and the recipient’s ileal and colonic samples had alpha diversity indices > 3.0, on the date of transplant. During the first endoscopy, although the alpha diversity index of the recipient’s ileal sample dropped to 1.59, the sample contained a large fraction of Bacteroidetes (26.3%) and Firmicutes (65.4%). During the second endoscopy, the alpha diversity indices of the samples increased (to 2.95), with expansions observed in both dominant phyla to include diverse Bacteroidetes and Firmicutes.

### Antimicrobial resistance

**Figure 3.**
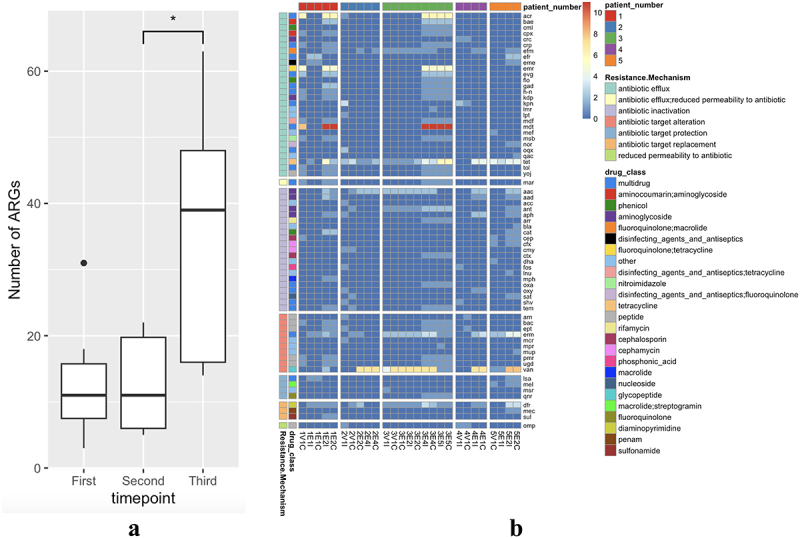
Quantification of antimicrobial resistance genes (ARG) present in participant samples.

Post-operatively, all recipients were heavily exposed to antimicrobial agents as shown in Table 2 and described in supplementary data. Figure 3 shows the temporal variations in the carriage of AMR genes in SBT participants. There was no significant difference between the total number of AMR genes detected when comparing samples taken at time point 1 (day of transplant) and time point 2 (first endoscopy), with a median of 11 AMR genes detected at both timepoints. In contrast, there was a significant increase (*p* < 0.05) in the number of AMR genes detected between the second (first endoscopy) and third (second endoscopy) time points (Figure 3A). Importantly, we did not detect any AMR genes in donor samples collected on the day of transplantation.

A) Cumulative number of ARGs in all patient samples at time point 1 (day of transplant,) time point 2 and 3 (1st and 2nd endoscopy follow up.) *Significant difference in number of ARGs between timepoints. B) ARG evolution in samples, over time, by participant. ARG are grouped by mechanism of resistance and annotated by antimicrobial drug class to which they confer resistance. If an AMR gene conferred resistance to ≥ 3 antimicrobial classes, the antimicrobial drug class was classified as ‘multidrug’ resistance.

**Table 2.** Clinical summary of participants.

Participant	Age	Transplanted organs	Past MDRO*	Antimicrobial treatment	Immunosuppressive maintenance	Toxo IgG status (D/R)	Pathogens	Sepsis or rejection episode?	Other infection related complications?	Survival at 1 year	Length of stay (days)
1	52	Small bowel, colon, pancreas	None	Meropenem Vancomycin Caspofungin	Tacrolimus Methylprednisolone Mycophenolate mofetil	-/-	*Candida glabrata*	Sepsis	None	Alive	53
2	29	Small bowel, stomach, colon, pancreas	None	Meropenem Metronidazole Ciprofloxacin Vancomycin Tigecycline Amikacin Cidofovir Anidulafungin	Tacrolimus Methylprednisolone Mycophenolate mofetil Thymoglobulin (rabbit anti-thymocyte globulin)	-/-	*Enterococcus faecium*	Sepsis & Rejection	Delirium: Fall with subdural hemorrhage.	Died	164
3	43	Jejunum, colon, pancreas, liver	Vancomycin resistant *Enterococcus faecium*	Daptomycin Metronidazole Gentamicin Vancomycin Ciprofloxacin Tigecycline Voriconazole Linezolid	Tacrolimus Methylprednislone Mycophenolate mofetil	-/-	*Aspergillus fumigatus*	Sepsis	Readmission with fungal discitis.	Alive	144
4	24	Small bowel, pancreas, liver	None	Vancomycin	Tacrolimus Methylprednislone	-/-	*Enterococcus faecium* *Klebsiella michiganensis* *Enterococcus faecium*	None	Intra-abdominal bleed: Mycotic breakdown of aortoconduit anastomosis.	Died	18
5	43	Intestine, pancreas, liver	None	Meropenem Fluconazole Ertapenem	Tacrolimus Methyprednisolone Mycopheolate mofetil	-/-	None	None	Abdominal collection and chyle leak. Pyrexia of Unknown Origin.	Alive	57

*MDRO- Multi-drug resistant organisms.

There was a high abundance of the *van* gene (Figure 3B) (known to confer vancomycin resistance in *Enterococcus* species) detected in participants 2,3,4 and 5, along with *mdt* and *acr* genes, which encode multi-drug efflux pumps. The tetracycline efflux pump encoded by the *tet* gene was detected in all participants. Among the acquired AMR genes, trimethoprim (*dfr*), aminoglycoside (*aac*), macrolide, lincosamide, and streptogramin (*erm*) were detected in all five participants. Trimethoprim-sulfamethoxazole is commonly used as universal prophylaxis after SBT and solid organ transplantation from day 14. Both *erm* and *aac* genes are frequently carried by *Enterococcus faecium*.^41^ We did not observe a significant negative association between alpha diversity at the species level and AMR carriage in our limited cohort.

### Bacterial functional metabolic pathways

**Figure 4.**
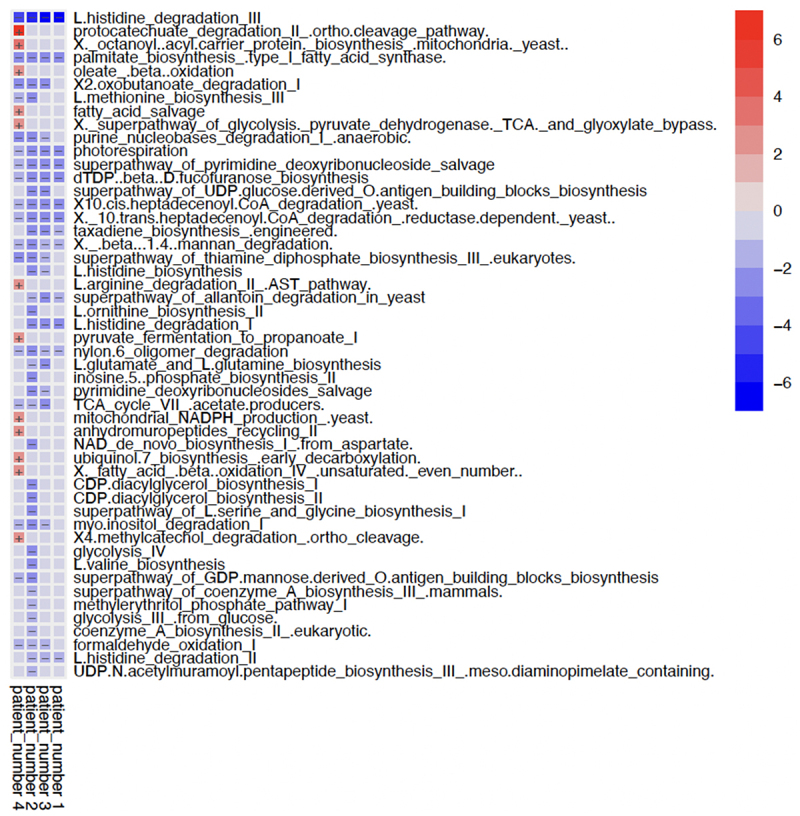
Multivariable association matrix depicting the top 50 functional pathway genes with significant alterations.

In this small SBT cohort, the functional bacterial metabolic pathways were differentially affected (Figure 4). Significantly higher levels of specific metabolic genes were exclusive to Participant 4. Participant 1, who also had a good clinical outcome, had the least significantly altered functional pathway genes compared to participant 5. Seven pathways, the majority of which are involved in glucose and lipid metabolism, were found in significantly lower abundance across participants 1–4 when compared to participant 5.

Metabolic gene abundances are represented in participants 1, 2, 3 and 4, in relation to participant 5. Participant 5 had the best post-op outcome: No sepsis or rejection episodes, survival at 1 year and short hospital in-patient stay. Red depicts significantly enriched metabolic pathway genes and blue depicts significantly depleted genes.

### Ileum and colonic gut microbiome composition

**Figure 5.**
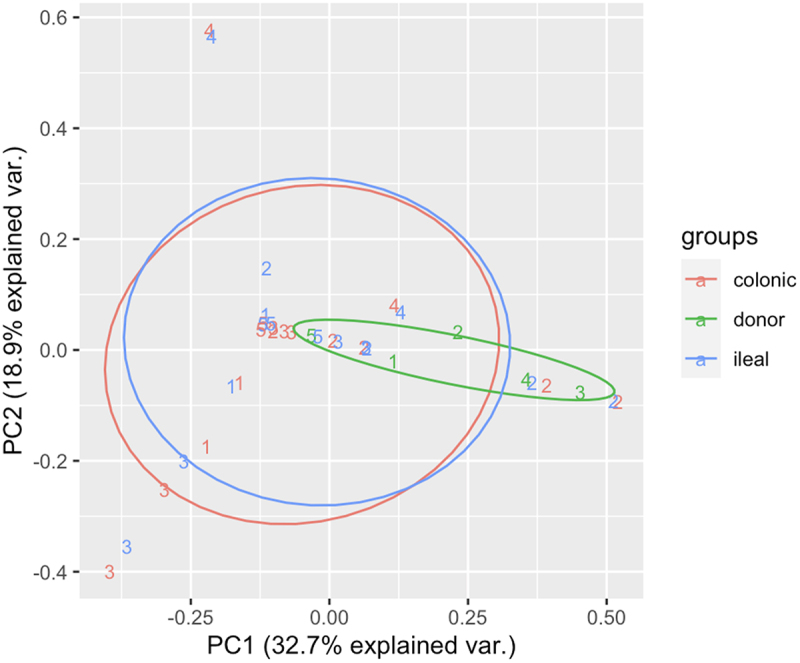
Principal coordinate analysis depicting the microbial composition of the three sample types: donor, recipient ileum and recipient colon.

Principal coordinate analysis revealed the microbial composition of the donor, recipient ileum, and recipient colon samples. The ileal and colonic microbial compositions of the transplant recipients were concordant, as shown in Figure 5.

Ellipses show a large overlap in the ileal and colonic microbial compositions in the transplant recipients.

### Linking isolates from clinical infection episodes back to the gut microbiome

Selected bacterial samples (marked with an asterisk in Table 1) underwent whole-genome sequencing. These were: participant 2’s VRE from a bacteremia episode at the end of their hospital stay, participant 3’s VRE isolated from body fluid, and Participant 4’s *Klebsiella michiganensis* isolated from peritoneal fluid. These organisms were present at high relative abundances in the gut microbiome prior to causing clinical infection.

InStrain analyses showed that all three of these isolates could be linked back to the gut microbiomes of their respective patients (highlighted in Supplementary data, Table 2). Patient 3’s VRE isolate mapped to their effluent samples at the second follow-up endoscopy (3E2I, 3E2C) with a population-level ANI (popANI) of 100% and zero single-nucleotide polymorphisms (SNPs). Patient 4’s *Klebsiella* michiganensis isolate mapped to their first endoscopy samples (4E1I, 4E1C) with nearly 100% coverage overlap, 99.99% popANI and 1 SNP. Finally, Patient 2’s VRE isolate mapped to their fourth endoscopy samples (2E4I, 2E4C) with 99.99% popANI and 3 SNPs. Moreover, Patient 2 and Patient 3’s VRE isolates appeared to be similar at the strain-level (Supplementary figure s2), since both isolates mapped with gut microbiome samples from patients 2, 3 and 4 with high ANI values (99.9–100%), and 0–12 SNPs. This finding was confirmed with a FastANI value of 99.78 between the two VRE isolates and a MASH distance of 0.0026, indicative of shared sequence-type. MLST was performed *in-silico* to identify the exact sequence-type, which classified both isolates as ST80 *Enterococcus faecium*.

## Discussion

This prospective cohort study used WMS to assess longitudinal variations in the intestinal microbiota of five SBT recipients at a major UK adult transplantation center. Our primary objective was to show a temporal shift in microbiota composition with respect to clinical infection and confirm our hypothesis that gut colonization can precede invasive infection by the dominant species. To extend this objective, we used WGS on isolates recovered from clinical infection episodes and attempted to map their genomes to the metagenomic bowel effluent data to assess bacterial translocation from the gut. We met our secondary objectives, reporting the changes after SBT of 1) AMR carriage and 2) genes encoding functional metabolic pathways in the gut bacteria. Overall, this study shows ecological shifts of the human microbiome in response to SBT, under the pressure of antimicrobials and immunosuppressive therapy, and establishes a baseline to measure the effects of future therapeutics.

### Comparison to previous SBT cohorts

Our cohort was similar to global SBT cohorts in terms of age, indications for transplantation, and percentage of liver-containing transplantation, which are known to be associated with improved graft survival (but lower overall patient survival).^4,12,42^ Our cohort’s relatively low survival rate at 1 year of 60% likely reflects our small group size. The rejection rate of 20% and sepsis rate of 60% in our cohort were comparable to previously published data.^5,12^ The organisms causing abdominal infections in our cohort are commonly observed in SBTs.^7,43,44^

### Pathogens outcompete the commensal microbiota before causing clinical infection

Many studies have suggested that the gut microbiome plays a critical role in patient outcomes following SBT. Approximately 90% of the 100 trillion members of the gut microbiome in healthy adults comprise two phyla: Bacteroidetes and Firmicutes,^45^ which were present at high relative abundance in all five samples from participant 5. During surgery, exposure of the bowel lumen to oxygen alters the microbial community structure from consisting primarily of obligate anaerobes, such as *Bacteroides* and *Clostridia* to facultative anaerobes, such as *Lactobacilli* and *Enterobacteriaceae*.^11^

In our small but otherwise typical SBT cohort, we observed an increased abundance of specific pathogens in the gut (*Enterococcus faecium*, *Klebsiella* spp., and *Candida glabrata*) before the onset of clinical infection. Furthermore, we identified that three isolates recovered from clinical infection episodes were either closely genetically related or identical sequence types to the abundant taxa in the gut microbiota. These microorganisms had colonized the gut at clinically meaningful intervals before manifesting as the apparent causes of clinical disease. This interval allows for the possibility of therapeutic intervention. Such interventions could involve targeted antimicrobial treatment or fecal microbiota transplantation (FMT), which have previously been employed in transplant patients colonized with multi-drug resistant (MDR) bacteria.^46^ FMT has been successfully used in renal transplant patients (colonized with ESBL-producing *E.coli*) and renal/heart transplant patients (colonized with *Clostridium difficile* and VRE) to prevent subsequent clinical infections with these MDR organisms.^47^

A previous study showed that microbiome changes in SBT recipients are temporally associated with the risk of infection as well as rejection episodes.^16^ VRE, MDR, and extensively drug-resistant (XDR) *Enterobacteriaceae* and MDR/XDR non-fermenting gram-negative bacteria are increasingly recognized as common causes of infection in solid organ transplant patients.^43^ Notably, expansions of the *Enterococcus* and *Klebsiella* genera and low pre-transplant alpha diversity in the gut have been associated with an increased risk of developing MDR infections in liver transplant recipients,^48^ which could explain the rapid deterioration post-multi-visceral SBT in participant 4 in our study. Larger longitudinal metagenomic studies in SBT patients are required to ascertain the range of organisms capable of gut translocation, which could enable targeted screening strategies in the future.

### Gut microbiota composition and acute rejection

A historic cohort study of 19 SBT patients showed differences in the microbiome of patients with and without acute graft rejection using 16S sequencing data, and suggested that a low abundance of Firmicutes and a high abundance of Proteobacteria could be associated with rejection.^49^ Our study adds to the existing literature by offering insight into the impact of SBT on the microbiome and subsequent rejection and sepsis episodes in the immediate post-operative period with species-level resolution using metagenomic data. While we also found a high abundance of Proteobacteria in participants with complications post-SBT, we only described the clinical course of one participant with recurrent rejection episodes (Participant 2).

### Carriage of enterococcus faecium and risk of infection

An increase in the order Lactobacillales has previously been proposed as a reliable marker for acute rejection.^49^ However, our findings are in contrast, with four out of five participants in our study showing notable abundance of *Enterococcus faecium*, belonging to the order Lactobacillliales. Three of four VRE-colonized participants in our study developed clinically significant infections. Here, colonization status (both pre- and post-transplant) appeared to be a stronger predictor of post-transplant bacteremia, which aligns with previous reports of VRE infections in solid organ transplant recipients colonized with VRE.^50,51^ While this discrepancy could be attributed to our small sample size, the two studies recruiting patients of different age groups (predominantly pediatric vs. adults) or distinct geographical regions (USA vs. UK), with frequent reports of VRE in our institution,^52^ it could also signal a transition toward an increase in nosocomial VRE in the UK.^53^ The two VRE isolates we recovered and sequenced belonged to ST80, a highly-prevalent MDR strain which has been reported across Europe.^52,54,55^

A previous study on SBT showed that post-transplant colonization with VRE is associated with a nine-fold increased risk of developing bacteremia compared to non-colonized hosts (36% versus 4%), but this did not translate into differences in one-year survival.^51^ Collectively, these findings highlight the challenges of generalizing at higher taxonomic levels, limited by the resolution of the 16S microbiome data used in previous microbiome studies in SBT cohorts. As sequencing technologies become increasingly affordable, we emphasize the need for more comprehensive genus/species-level observations using whole-metagenome data for SBT patients across demographic strata.

### Higher alpha diversities are associated with better outcomes

In this study, we noted that recipients with better outcomes received transplanted donor samples with higher alpha diversity. Through its competitive exclusion ability, a diverse gut microbiome can outcompete existing or introduced pathobionts and prevent them from colonizing the host.^56^ In addition, mixed microbial populations in the gut create redundancies in key metabolic pathways, thereby permitting compensatory means of producing essential metabolic intermediates for healthy gut function.^57^ We also observed that non-survivors in our cohort had alpha diversities below 2.5 at their final time point. These findings need to be replicated in larger studies to explore the possibility of donor selection based on alpha diversity in addition to the potential for post-operative surveillance of recipient gut microbiomes to monitor recovery. Beta-diversity was highly varied between samples taken from the same participant at different time points (data not shown), which contrasts findings in healthy subjects^58^ and potentially reflects the effects of the antimicrobial and immunosuppressive therapy patients received in the post-operative period.

### AMR correlates with duration of inpatient stay

AMR is an emerging problem in transplanted patients.^59^ Intensive antimicrobial use alters the gut microbiome and promotes colonization by MDR bacteria.^19,60,61^ Recent guidelines for the management of MDR infections in transplant recipients call for studies to assess colonization using WGS to understand antimicrobial impact and the association between colonization and infection.^62^ Our study provides preliminary metagenomic data in SBT recipients that could help inform strategies to tailor antimicrobial plans for individuals to lengthen the time to first bloodstream infection without encouraging the development of further AMR.^63^

In the present study, there was an increase in the abundance and diversity of AMR genes over time. We suggest that this could be partially due to the timing of our sampling, which was conducted during a period of heavy immunosuppression and initiation of broad-spectrum antimicrobials in a hospital setting. The association with the length of hospitalization underlines the importance of early discharge and removal from a potentially contaminated hospital setting when possible. In this small study, we saw no AMR gene carriage in the donor grafts, despite previous studies suggesting that a healthy gut environment can be a reservoir for AMR genes.^64^ We suspect that the absence of AMR gene detection despite adequate sequencing quality in donor samples can be attributed to the characteristics of individual donors. In the UK, deceased donors often suffer from cerebral vascular accidents and traumatic brain injury^65^, making them unlikely to be exposed to long courses of antimicrobials; therefore, there is limited selection pressure for AMR development in donor gut microbiomes.

### Metabolomic exploration

The gut microbiota is known to modulate intestinal homeostasis by aiding energy metabolism through the exportation of key microbial metabolic products, such as short-chain fatty acids (SCFAs), amino acids, and vitamins.^66,67^ SCFAs such as acetate, propionate, and succinate constitute 10% of the daily caloric requirements of humans as important energy substrates for colonocytes and regulators of lipid and carbohydrate metabolism.^68^ For example, Gram-negative *Veillonella* (Firmicutes) and *Parabacteroides* (Bacteroides), found in high abundance in participants 1 and 5 respectively (Figure 1), are involved in the production of propionate and succinate which activate gluconeogenesis in the liver, and have been implicated in gut recovery.^69^ The genes associated with the histidine degradation pathway, which produces histamine, were also found to be more abundant in participant 5. Microbiota-derived histamine from *Lactobacillus* spp. modulates the immune response by suppressing the pro-inflammatory effects of TNF-α in a host receptor-dependent manner.^70,71^ It is also noteworthy that participants 1, 3, and 5 had a high abundance of *Lactobacillus* spp. (Supplementary Data, Figure s1) and were all alive at 1-year. In addition, genes involved in the fatty acid beta-oxidation pathway (found in high abundance in participant 4) encode a restorative mechanism to maintain anaerobic conditions and prevent facultative anaerobes from proliferating and infiltrating the gut lumen upon exposure to oxygen.^72^ As an emerging field, the true relevance of these and the remaining pathways detected in patient outcomes needs to be further validated in a larger study cohort with interdisciplinary collaborations to identify potential targets for intervention.

### Limitations

Our study has several limitations. Primarily because of the small sample size, our findings are descriptive. Since the study was conducted in the post-pandemic period, we received relatively fewer follow-up endoscopy samples as post-transplant endoscopy sampling was streamlined. Future studies with larger sample sizes are needed to overcome this limitation.

We accept this study cannot begin to evaluate donor-related factors known to impact gut microbiome. It was not possible to collect donor data (age, gender, smoking status, weight, diet, known carriage of resistant bacteria) due to ethical constraints. With the scarcity of organs for transplant, clinicians presently may not be able to await an organ block with optimal microbiome alpha biodiversity, even if this analysis could be performed in a timely manner. However, there may be some utility in performing this if it could be used to correlate with clinical outcomes or predict patients more at risk of adverse outcomes. Further studies with the necessary ethical approvals could be considered.

Sample size also impacts sequencing analyses due to variability in the number of raw reads per sample during sample collection. We set our sequencing threshold at 30,000 reads per sample and excluded three samples from alpha diversity, AMR, and metabolomic analyses due to inadequate sequencing depth (see Methods). A larger cohort would enable the sequencing threshold to be set at 45,000 reads per sample for community-level analyses without excluding significant sample numbers. The Kraken2 analysis pipeline also misclassified *Mycobacterium canetti* and *Toxoplasma gondii* due to recognized gaps in reference genomes and a tendency for the program to misclassify human DNA as *Toxoplasma gondii*.^73^ Additionally, the IgG pre-screen was negative for all five participants, which suggested false positive OTU assignments for the taxon. Therefore, these reads were removed from the downstream analyses. We were also unable to recover the isolate which caused VRE infection in participant 4, and therefore cannot validate the genetic similarity observed with the isolates recovered from participants 2 and 3, despite the data suggesting high ANI between these isolates and participant 4’s gut samples.

Finally, a larger sample size is required to thoroughly explore the impact of shifts in the metabolic potential of the microbiome on patient outcomes. Further steps would involve the investigation of correlations between metabolic pathway gene abundance and gene expression using RNA-seq. Thus, dysregulated pathways may be targets for future clinical trials that can translate knowledge in this area to promote the development of novel vaccines, monoclonal antibodies, FMT, targeted bacterial metabolic pathway alterations, and help generate microbiota-driven therapeutics.^47,74^ Despite these limitations, our study pioneers the use of WMS on longitudinal gut microbiota samples from SBT patients. We contribute new data to support the bacterial translocation hypothesis of gut-origin sepsis in patients undergoing intestinal transplantation and offer preliminary insights into AMR gene carriage and metabolic potential in the SBT cohort.

## Conclusion

This metagenomic study adds to the body of evidence supporting the gut-origin sepsis theory in a rare cohort of SBT recipients, providing a rationale for initiating a similar, full-scale clinical study. We found preliminary evidence linking colonization by specific gut pathogens with episodes of sepsis. Ileal and colonic samples, matched by time point and participant, were concordant, which suggests that bedside fecal sampling from the stoma site could be an effective surveillance tool for impeding sepsis with specific bowel-residing pathogens. We found an increase in AMR gene abundance and diversity with an increase in inpatient stay duration. Finally, we showed that higher alpha diversities (in both donor and recipient microbiomes) were predictive of improved patient outcome. To aid future efforts to combat the threat of AMR and mortality associated with infections in SBT, this study is the first to capture the complete metagenomic landscape of the gut microbiome in patients post-SBT.

## Supplementary Material

Revised_SBT_supplementary_data_18122023.docx

## Data Availability

The authors confirm that the data supporting the findings of this study are available in the article and its supplementary materials. The microbial WMS reads for 40 patient samples and WGS reads for 3 bacterial isolates have been deposited in the NCBI Sequence Read Archive under BioProject accession PRJNA1004534.
