# Supplementary material for: A metagenomic prospective cohort study on gut microbiome composition and clinical infection in small bowel transplantation
Source: Gut Microbes. 2024 Mar 4;16(1):2323232. doi: 10.1080/19490976.2024.2323232 (PMC10936650; doi:10.1080/19490976.2024.2323232)
Supplement: Revised_SBT_supplementary_data_18122023.docx [file KGMI_A_2323232_SM0206.docx]

**SUPPLEMENTARY DATA**

***Antimicrobial Prophylaxis and Immunosuppression***

Post-operatively all recipients were heavily exposed to antimicrobial agents as shown in Table 1. They received antimicrobial prophylaxis with intraoperative intravenous antibiotic piperacillin-tazobactam (participant 1, 2, 3 and 4) or meropenem (participant 5) dependent on allergy status. Participant 1 received additional antimicrobial intravenous vancomycin due to previous MRSA colonization. Post-transplantation, recipients continued these prophylactic antimicrobial agents for 7 days.

Recipients received antifungal prophylaxis with intraoperative intravenous, narrow-spectrum, antifungal caspofungin (participant 2 and 4) or broad-spectrum, antifungal Amphotericin B liposomal (participant 1, 3 and 5.) This was dependent on the participant’s risk stratification calculated by presence of pre-existing asthma lung pathology (asthma) or history of previous cigarette-use. Antifungals were continued for 14 days, before switching to a narrower spectrum antifungal, oral fluconazole, for participants 2, 3 and 5. Participant 2 required an additional 4 weeks of caspofungin for intra-abdominal infection. Life-long oral prophylaxis co-trimoxazole, effective against *Pneumocystis jirovecii* and *Toxoplasma gondii*, was commenced on day 14.

Antiviral intravenous ganciclovir was given for 7 days if donor or recipient were Cytomegalovirus seropositive (participant 3), to prevent primary infection or reactivation, before switching to oral valganciclovir for one year. If donor or recipient were Cytomegalovirus seronegative (participant 1, 2, 4 and 5) acyclovir was given instead.

Peri-operatively, all recipients’ immunosuppression induction was achieved with alemtuzumab subcutaneously and methylprednisolone intravenously. Post-transplant, immunosuppression maintenance used tacrolimus and methylprednisolone. At day 21, oral mycophenolate mofetil was commenced for all surviving participants. Additionally, participant 2 required intravenous ATG rabbit (thymoglobulin) for treatment of ongoing rejection episodes.

**Detailed Laboratory Methods: Buffers**

Lysis Buffer

| Step | Reagent | Concentration required | Dilution Buffer | Weight/volume | Volume of buffer (to make 500ml) | Aliquot |
| --- | --- | --- | --- | --- | --- | --- |
| 1 | Guanidine Thiocyanate (MW 118.16g/M) | 4M | 25mM Tris pH7 | 236.32g | 500ml | N/A |
| 2 | B-mercaptoethanol | 0.5% | Lysis buffer | 250μl | 50ml | 500 µl (for vials)  10 ml (for vial top-up) |
|  | Carrier RNA | 1:10000 |  | 5μl |  |  |

Wash buffer 1 (1M Guanidine Thiocyanate, 25mM Tris pH7, 10% ethanol)

| Step | Reagent | Concentration required | Dilution Buffer | Weight/volume | Volume of buffer (to make 500ml) | Aliquot |
| --- | --- | --- | --- | --- | --- | --- |
| 1 | Guanidine Thiocyanate (MW 118.16g/M) | 1M | 25mM Tris pH7 | 59.08g | 500ml | N/A |
| 2 | Ethanol | 10% | Wash buffer 1 | 50ml | 450ml | 12.5ml |

Wash buffer 2 (25 mM Tris pH 7, 70% ethanol)

| Step | Reagent | Concentration required | Dilution Buffer | Weight/volume | Volume of buffer (to make 500ml) | Aliquot |
| --- | --- | --- | --- | --- | --- | --- |
| 1 | Ethanol | 70% | 25mM Tris pH7 | 350ml | 150ml | 25 ml |

| **Sample ID** | **Sample Type** | **Patient Number** | **Time Point** | **Sampling Timepoint** |
| --- | --- | --- | --- | --- |
| 1E1C | Colonic | 1 | 2 | Follow-up |
| 1E1I | Ileal | 1 | 2 | Follow-up |
| 1E2C | Colonic | 1 | 3 | Follow-up |
| 1E2I | Ileal | 1 | 3 | Follow-up |
| 1V1C | Colonic | 1 | 1 | Date of transplant |
| 1V1I | Ileal | 1 | 1 | Date of transplant |
| 2E1C | Colonic | 2 | 2 | Follow-up |
| 2E2C | Colonic | 2 | 3 | Follow-up |
| 2E2I | Ileal | 2 | 3 | Follow-up |
| 2E3C | Colonic | 2 | 4 | Follow-up |
| 2E3I | Ileal | 2 | 4 | Follow-up |
| 2E4C | Colonic | 2 | 5 | Follow-up |
| 2E4I | Ileal | 2 | 5 | Follow-up |
| 3E5C | Colonic | 3 | 6 | Follow-up |
| 3E5I | Ileal | 3 | 6 | Follow-up |
| 2V1C | Colonic | 2 | 1 | Date of transplant |
| 2V1I | Ileal | 2 | 1 | Date of transplant |
| 3E1C | Colonic | 3 | 2 | Follow-up |
| 3E2C | Colonic | 3 | 3 | Follow-up |
| 3E2I | Ileal | 3 | 3 | Follow-up |
| 3E3C | Colonic | 3 | 4 | Follow-up |
| 3E3I | Ileal | 3 | 4 | Follow-up |
| 3E4C | Colonic | 3 | 5 | Follow-up |
| 3E4I | Ileal | 3 | 5 | Follow-up |
| 3V1C | Colonic | 3 | 1 | Date of transplant |
| 3V1I | Ileal | 3 | 1 | Date of transplant |
| 4E1C | Colonic | 4 | 2 | Follow-up |
| 4E1I | Ileal | 4 | 2 | Follow-up |
| 4V1C | Colonic | 4 | 1 | Date of transplant |
| 4V1I | Ileal | 4 | 1 | Date of transplant |
| 5E1I | Ileal | 5 | 2 | Follow-up |
| 5E2C | Colonic | 5 | 3 | Follow-up |
| 5E2I | Ileal | 5 | 3 | Follow-up |
| 5V1C | Colonic | 5 | 1 | Date of transplant |
| 5V1I | Ileal | 5 | 1 | Date of transplant |

**Supplementary table 1: Sample anonymisation reference table.** Participants were assigned a unique number (1 - 5). Samples were assigned a code comprising the participant's number, followed by the transplantation visit (V1) or endoscopy number (E1-E6) and the site from which the sample was taken (ileal-I, colonic-C, donor-D).

| **Genome** | **Sample1** | **Sample2** | **Coverage overlap** | **Compared bases count** | **consensus SNPs** | **population SNPs** | **popANI** | **conANI** | **percent compared** |
| --- | --- | --- | --- | --- | --- | --- | --- | --- | --- |
| 003VRE | 2E4C | 2E4I | 0.5117 | 110314 | 62 | 0 | 1 | 0.999 | 0.03662 |
| 003VRE | 3E2C | 3E2I | 0.4978 | 1172314 | 41 | 0 | 1 | 1 | 0.38914 |
| 004KOX | 4E1C | 4E1I | 1 | 6052768 | 83 | 1 | 1 | 1 | 0.99988 |
| 002VRE | 3E2C | 3E2I | 0.5099 | 1244856 | 73 | 3 | 1 | 1 | 0.40929 |
| 002VRE | 2E4C | 2E4I | 0.5281 | 76353 | 95 | 3 | 1 | 0.999 | 0.0251 |
| 002VRE | 4E1C | 4E1I | 0.9888 | 2625143 | 351 | 6 | 1 | 1 | 0.8631 |
| 003VRE | 4E1C | 4E1I | 0.9936 | 2742511 | 301 | 12 | 1 | 1 | 0.91036 |
| 002VRE | 2E4I | 4E1C | 0.2609 | 232207 | 310 | 106 | 1 | 0.999 | 0.07635 |
| 002VRE | 2E4I | 4E1I | 0.2603 | 232378 | 331 | 118 | 0.999 | 0.999 | 0.0764 |
| 002VRE | 2E4C | 4E1C | 0.243 | 261150 | 363 | 125 | 1 | 0.999 | 0.08586 |
| 002VRE | 2E4C | 4E1I | 0.2421 | 261243 | 366 | 127 | 1 | 0.999 | 0.08589 |
| 003VRE | 2E4I | 4E1C | 0.2916 | 310918 | 345 | 172 | 0.999 | 0.999 | 0.10321 |
| 003VRE | 2E4I | 4E1I | 0.2915 | 311071 | 345 | 173 | 0.999 | 0.999 | 0.10326 |
| 002VRE | 2E4I | 3E2I | 0.3082 | 133380 | 299 | 212 | 0.998 | 0.998 | 0.04385 |
| 003VRE | 2E4C | 4E1C | 0.2836 | 327499 | 411 | 220 | 0.999 | 0.999 | 0.10871 |
| 003VRE | 2E4C | 4E1I | 0.283 | 327798 | 417 | 225 | 0.999 | 0.999 | 0.10881 |
| 002VRE | 2E4C | 3E2I | 0.2915 | 144833 | 366 | 258 | 0.998 | 0.997 | 0.04762 |
| 003VRE | 2E4I | 3E2I | 0.3569 | 159065 | 387 | 300 | 0.998 | 0.998 | 0.0528 |
| 003VRE | 2E4C | 3E2I | 0.3483 | 160110 | 418 | 345 | 0.998 | 0.997 | 0.05315 |
| 002VRE | 2E4I | 3E2C | 0.2347 | 215822 | 743 | 594 | 0.997 | 0.997 | 0.07096 |
| 002VRE | 2E4C | 3E2C | 0.2191 | 244589 | 813 | 646 | 0.997 | 0.997 | 0.08042 |
| 003VRE | 2E4I | 3E2C | 0.2745 | 257522 | 887 | 725 | 0.997 | 0.997 | 0.08548 |
| 003VRE | 2E4C | 3E2C | 0.2611 | 265112 | 950 | 810 | 0.997 | 0.996 | 0.088 |
| 003VRE | 3E2I | 4E1C | 0.461 | 1106690 | 2650 | 2418 | 0.998 | 0.998 | 0.36736 |
| 003VRE | 3E2I | 4E1I | 0.4605 | 1106911 | 2691 | 2423 | 0.998 | 0.998 | 0.36743 |
| 002VRE | 3E2I | 4E1C | 0.4533 | 1117851 | 3008 | 2680 | 0.998 | 0.997 | 0.36753 |
| 002VRE | 3E2I | 4E1I | 0.4531 | 1120937 | 3034 | 2706 | 0.998 | 0.997 | 0.36854 |
| 003VRE | 3E2C | 4E1C | 0.9306 | 2462334 | 7838 | 7296 | 0.997 | 0.997 | 0.81735 |
| 003VRE | 3E2C | 4E1I | 0.9303 | 2464500 | 7935 | 7346 | 0.997 | 0.997 | 0.81807 |
| 002VRE | 3E2C | 4E1C | 0.9212 | 2461207 | 8258 | 7577 | 0.997 | 0.997 | 0.8092 |
| 002VRE | 3E2C | 4E1I | 0.9227 | 2469144 | 8389 | 7690 | 0.997 | 0.997 | 0.81181 |

**Supplementary table 2: InStrain GenomeWide comparison table.** Cases where isolates mapped with high ANI to their host gut metagenomes are highlighted in green. Instances where VRE isolates mapped to alternative-host gut metagenome samples are highlighted in yellow.

**Supplementary figure 1: Pavian Plots by Participant Sample**

***Participant 1***


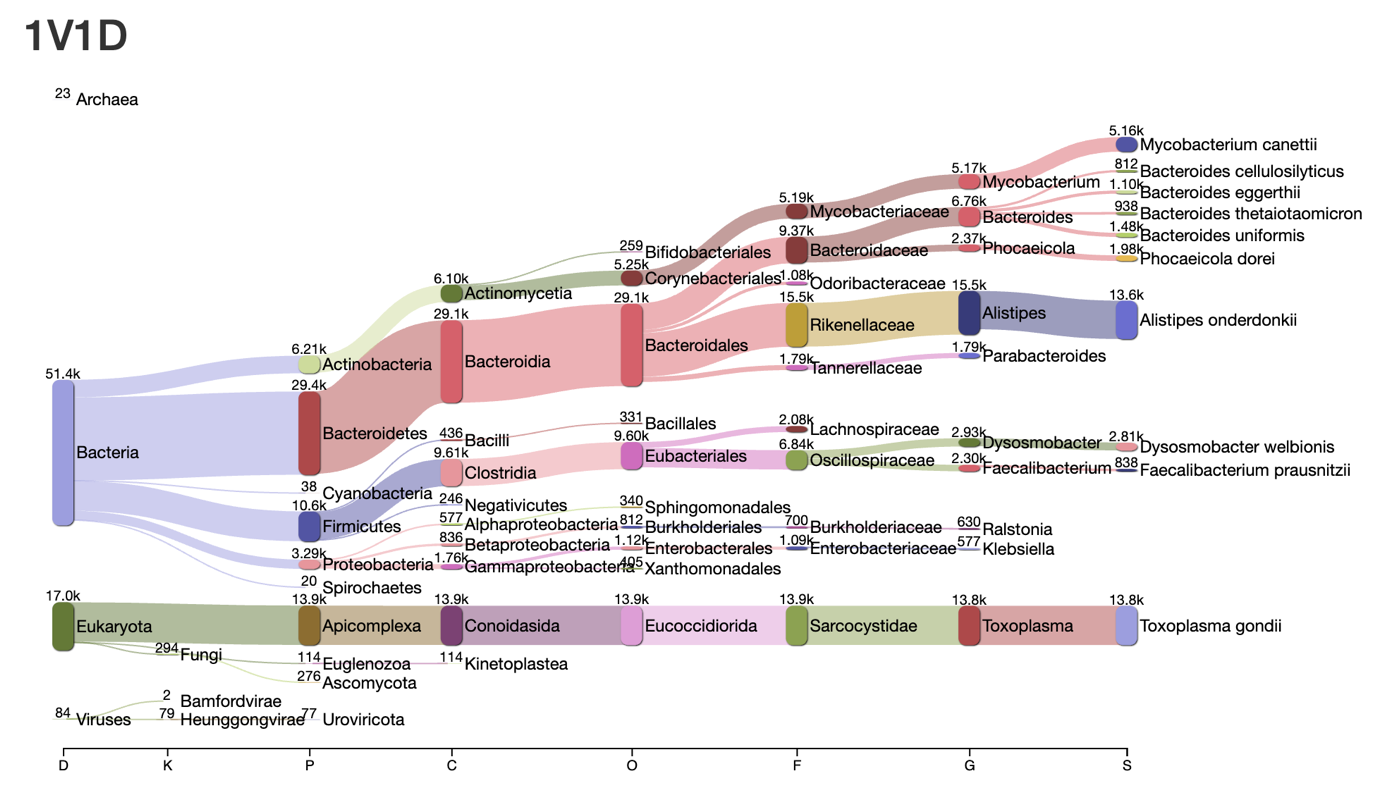


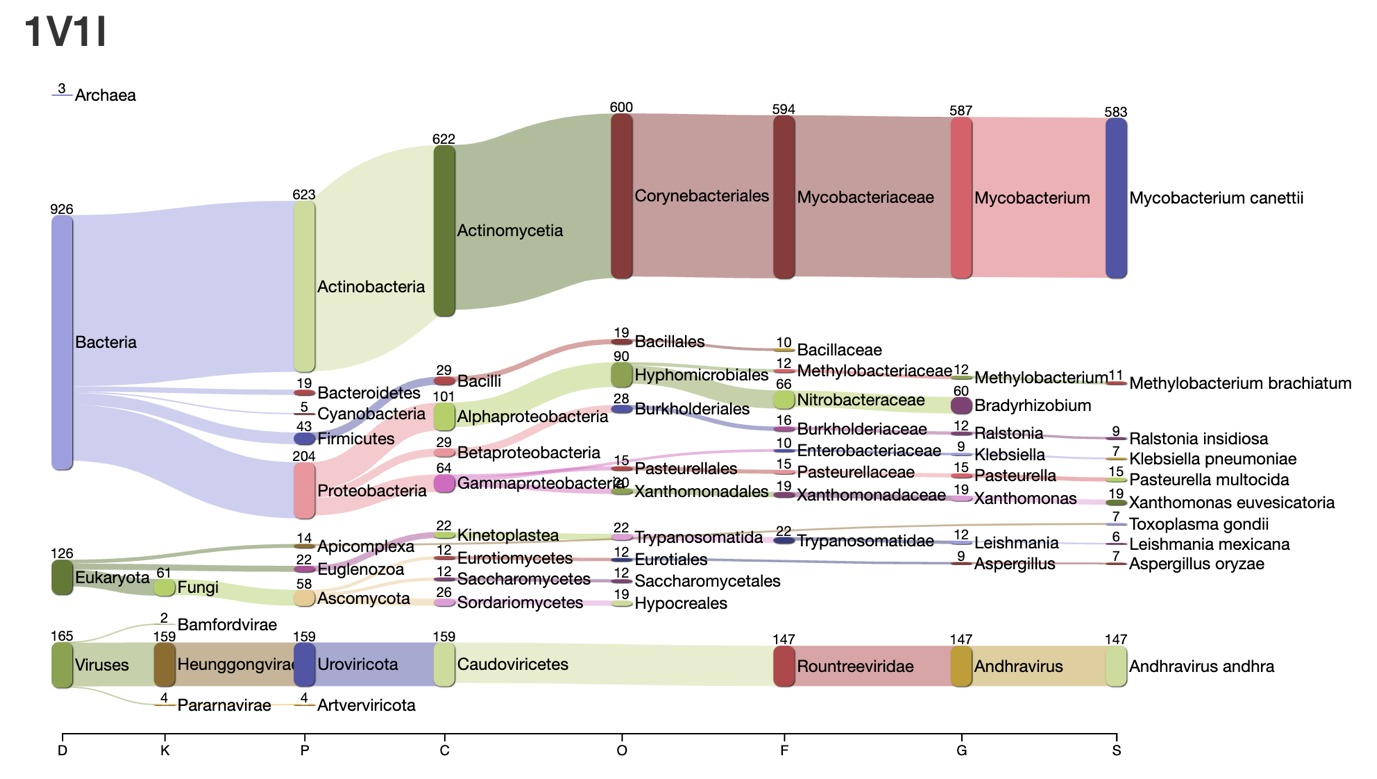


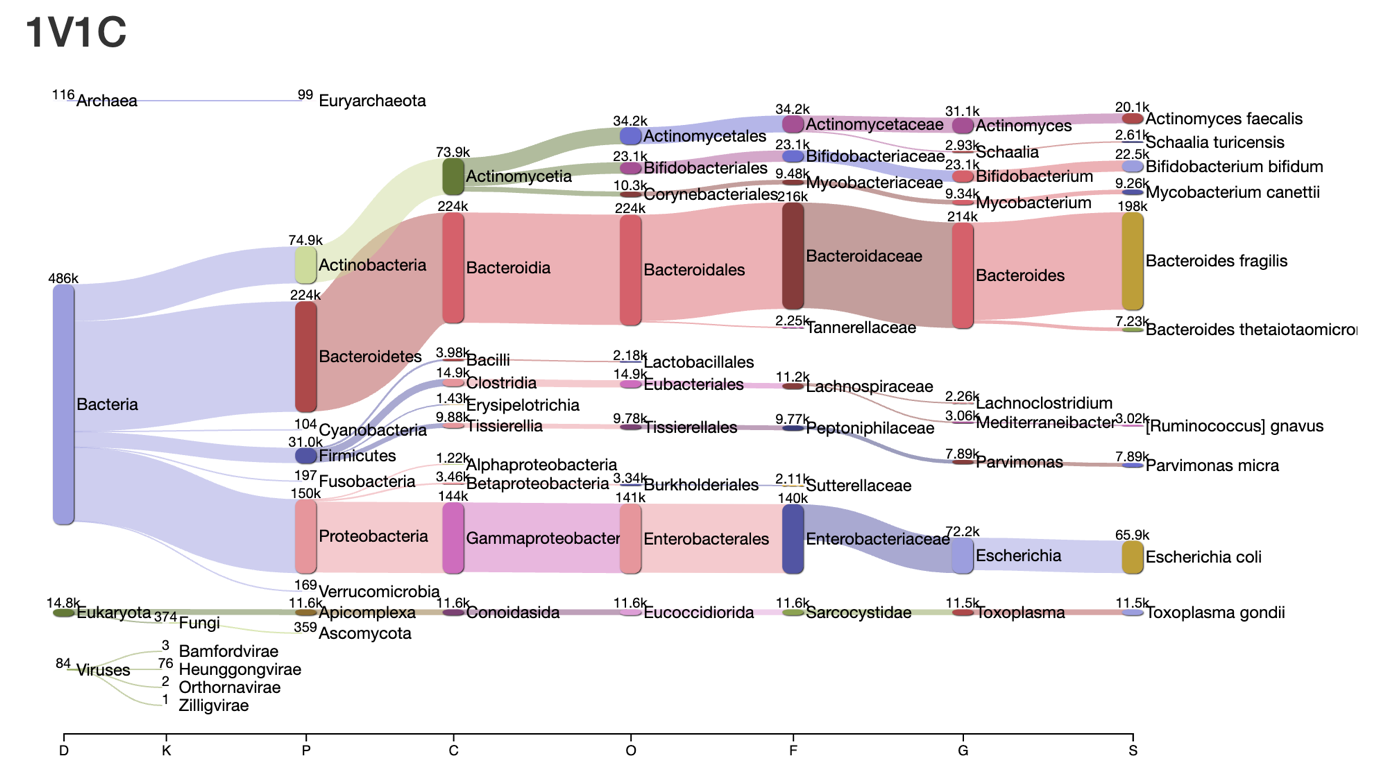


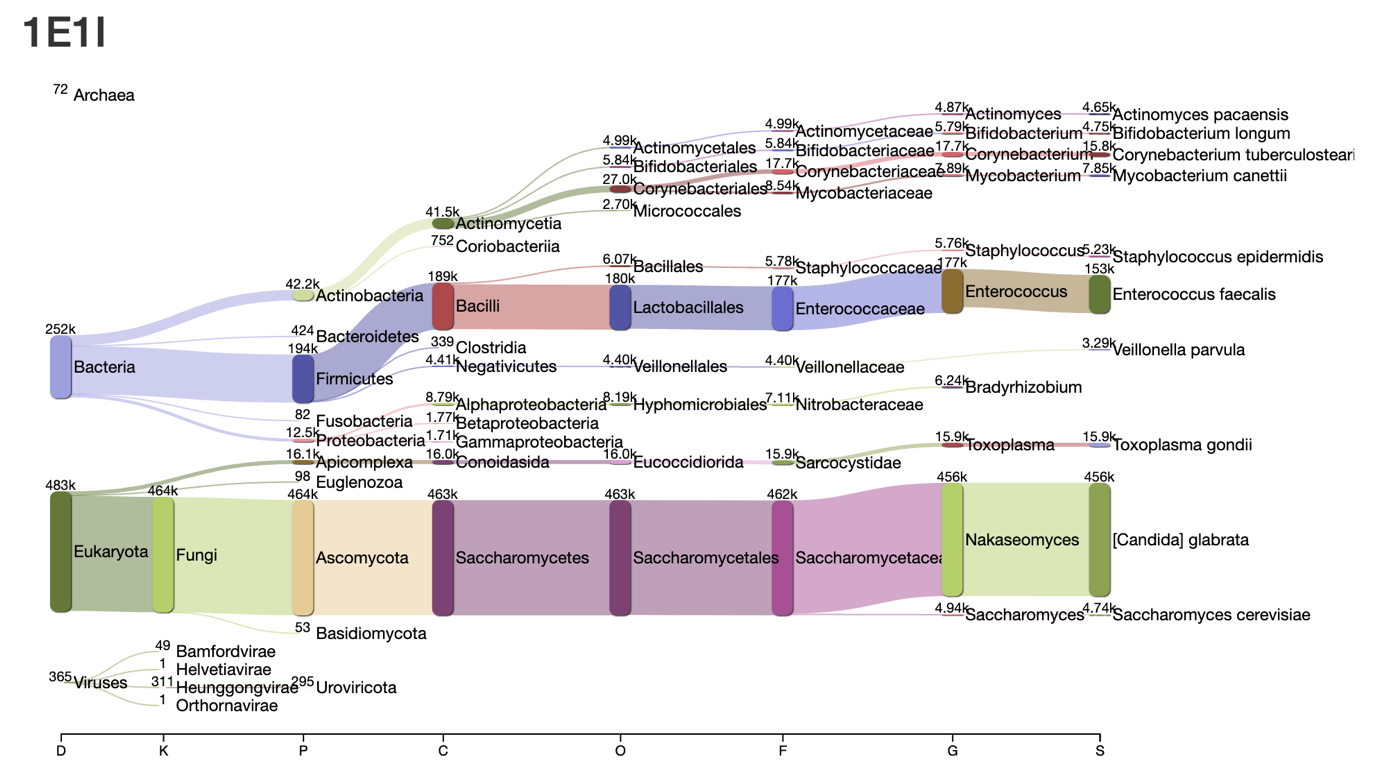


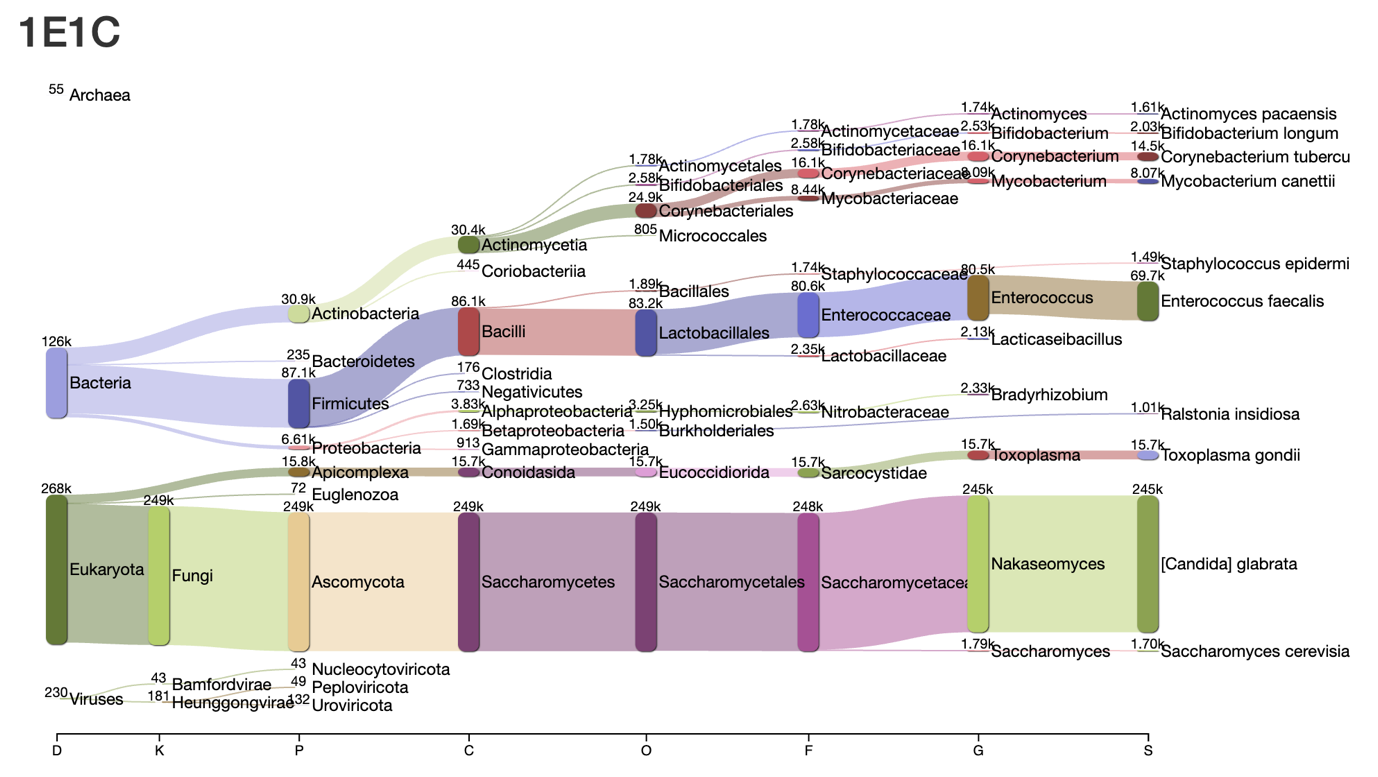


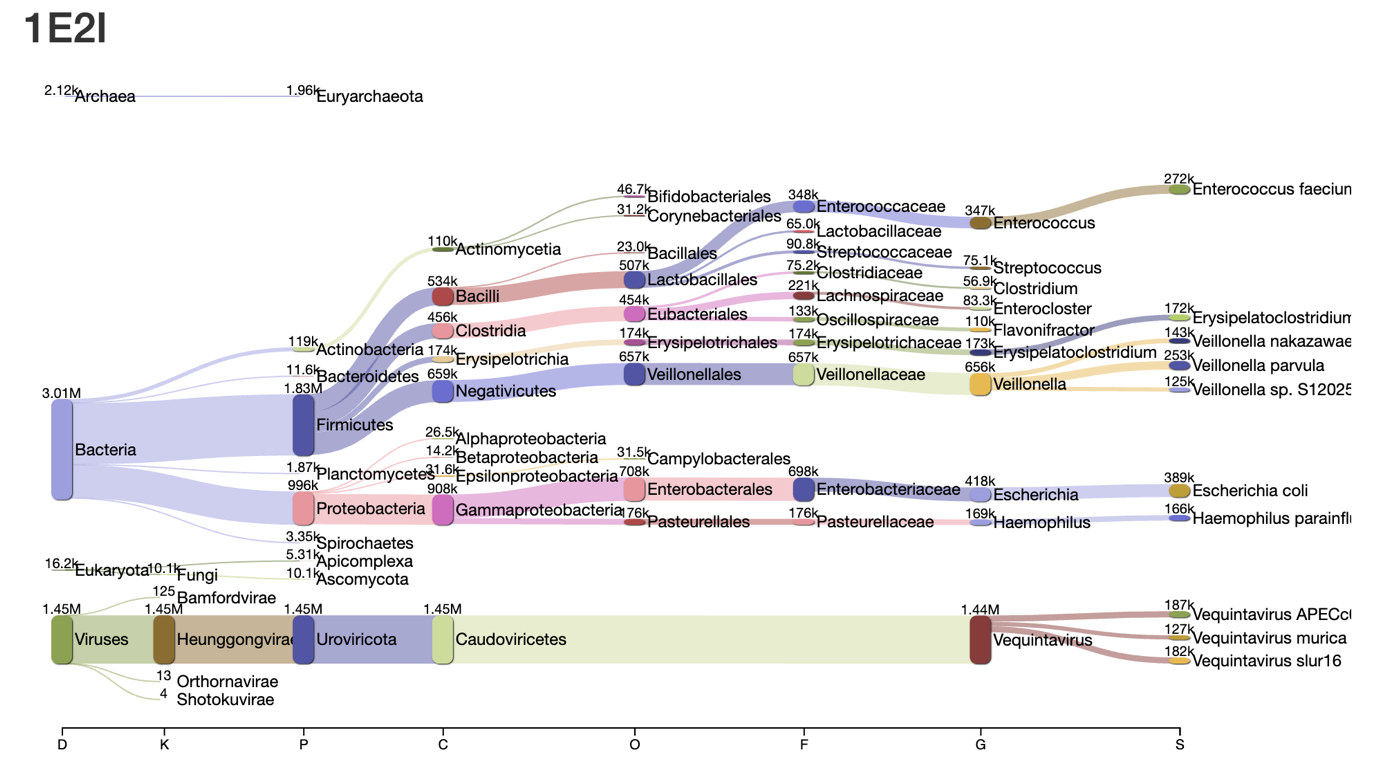


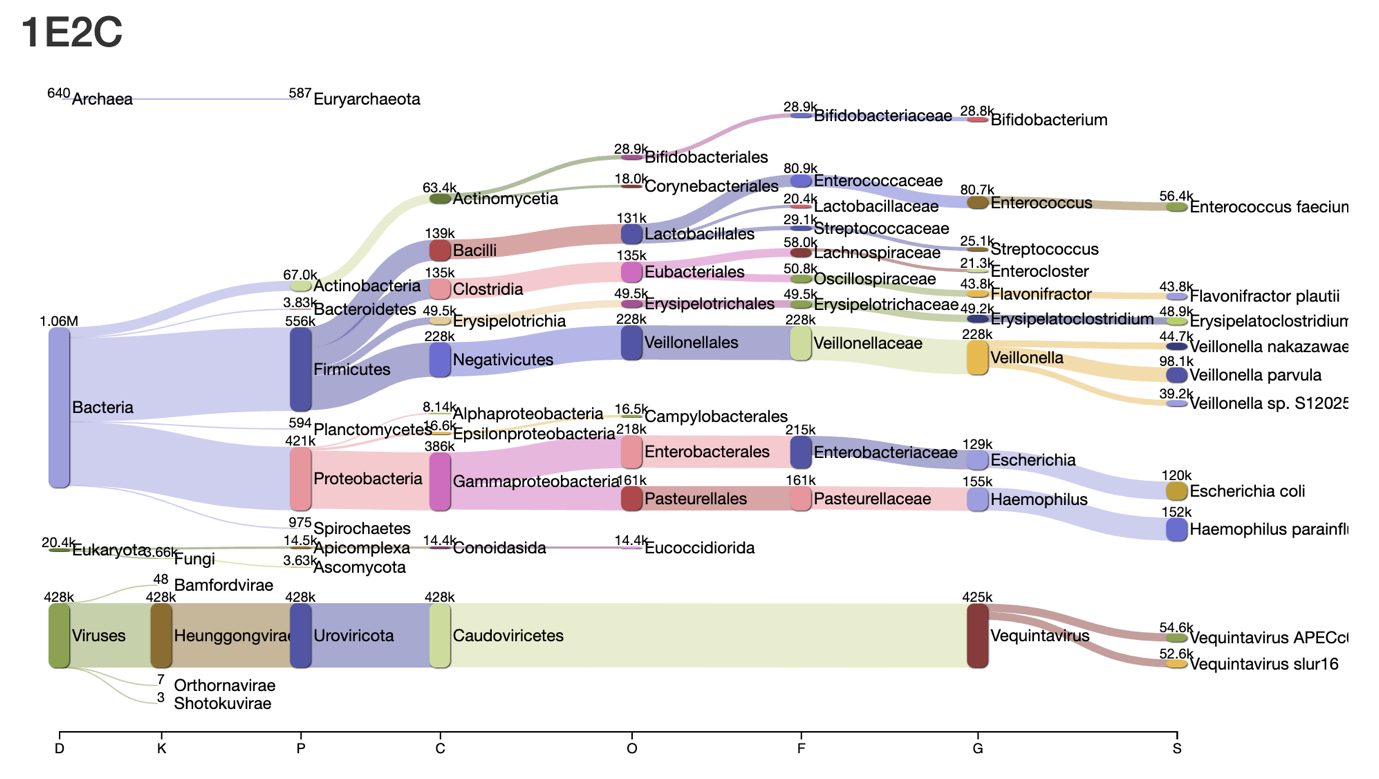


***Participant 2***


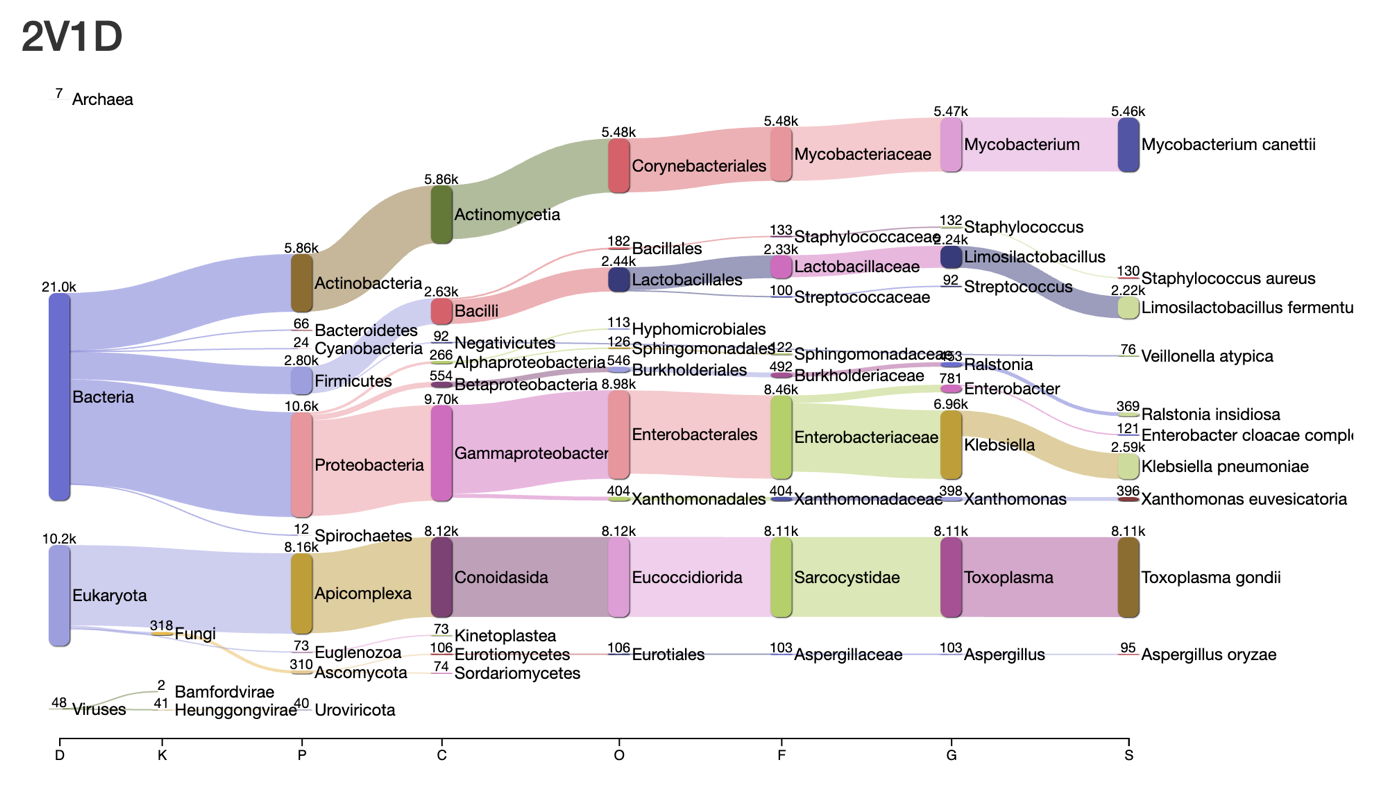


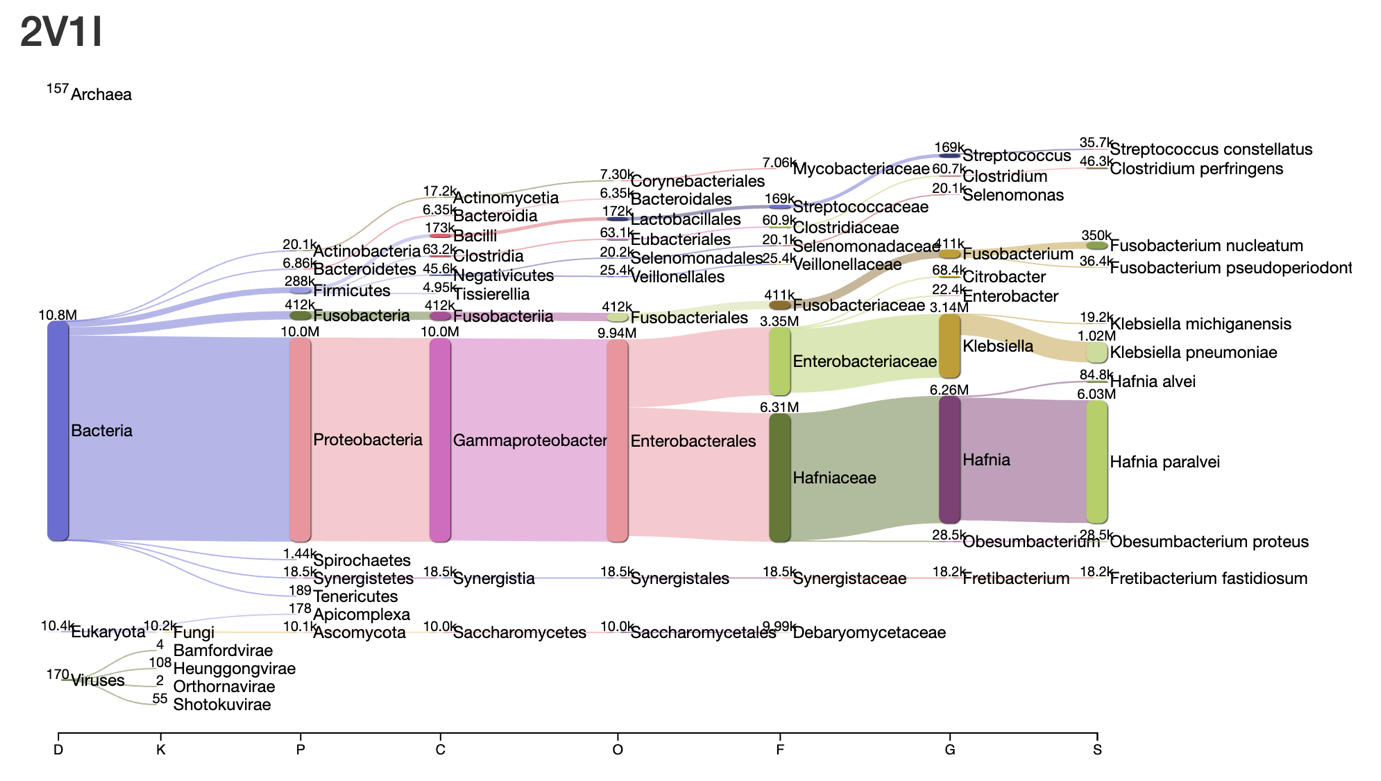


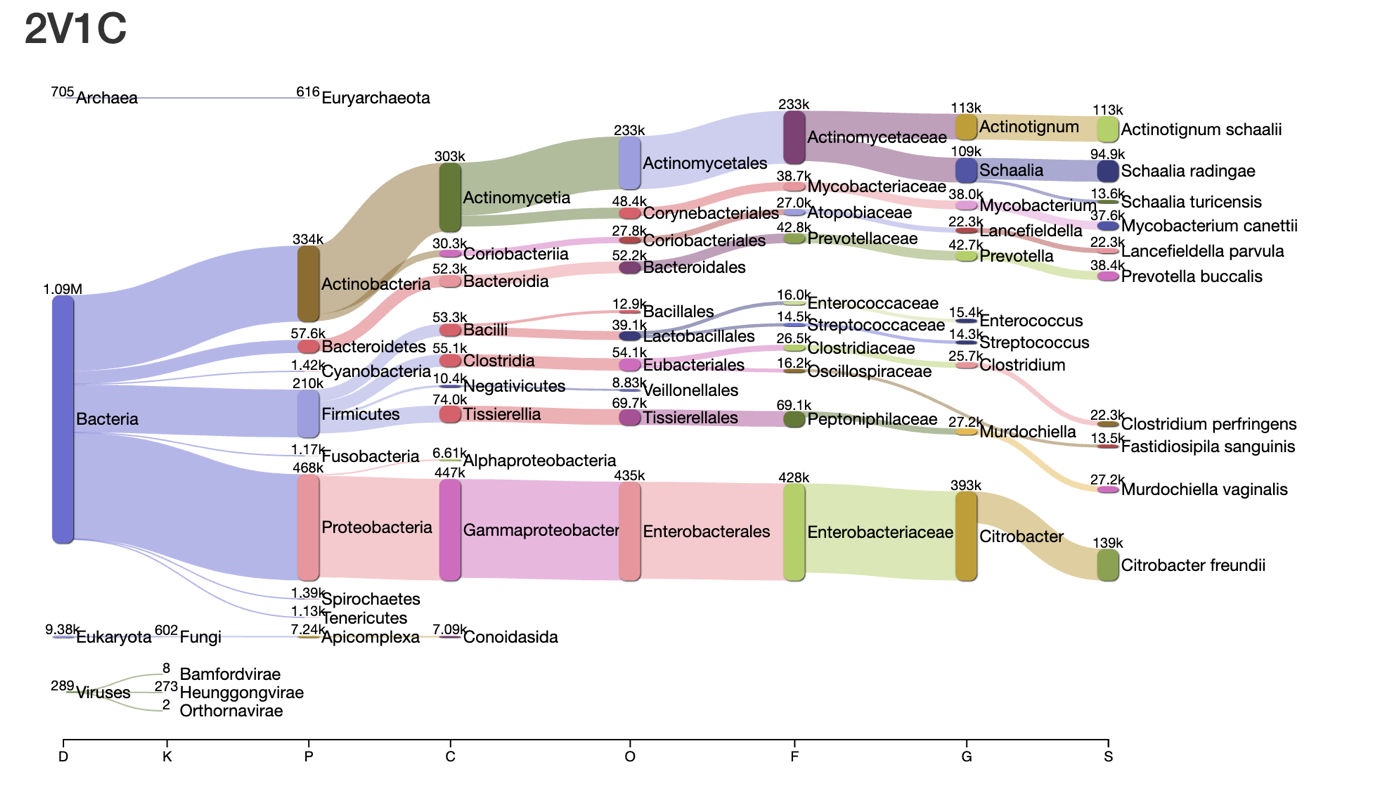


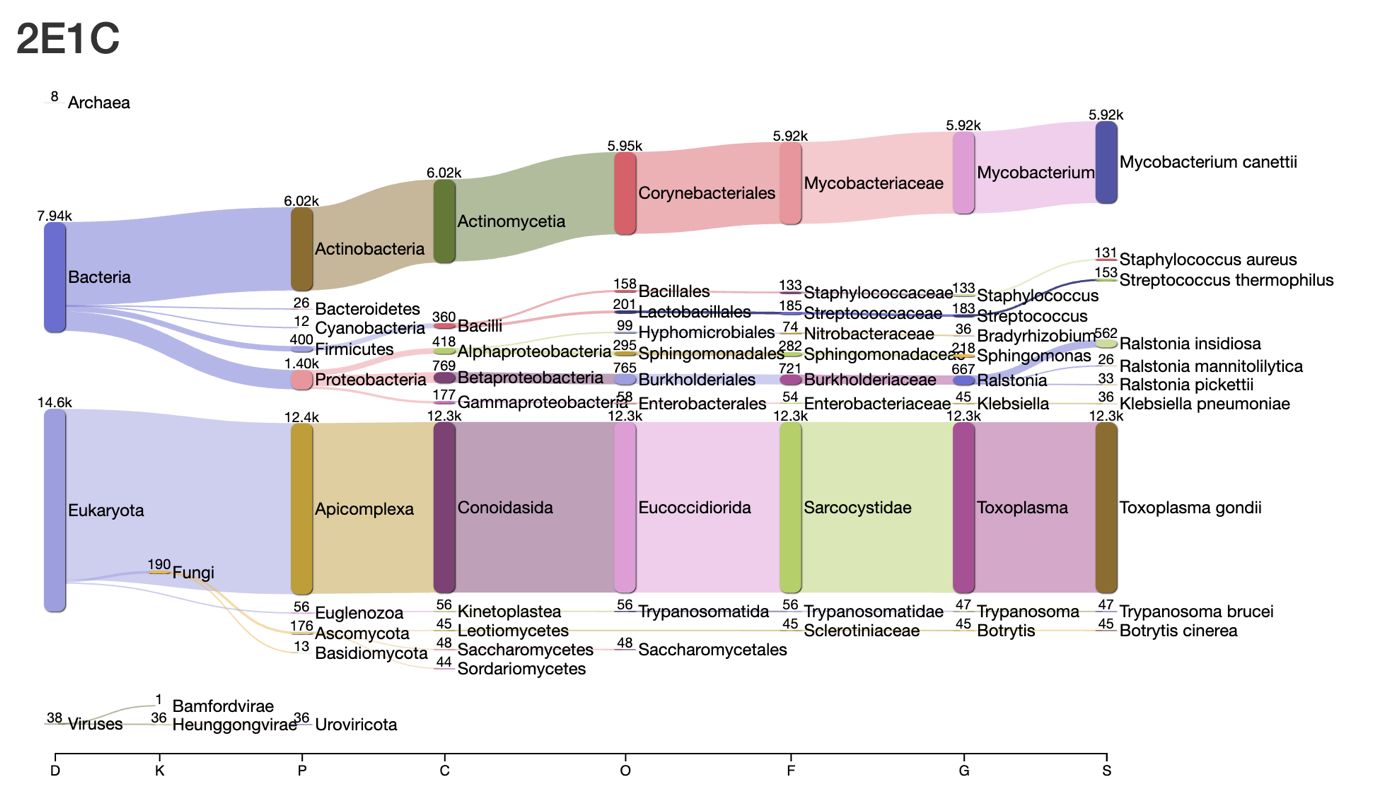


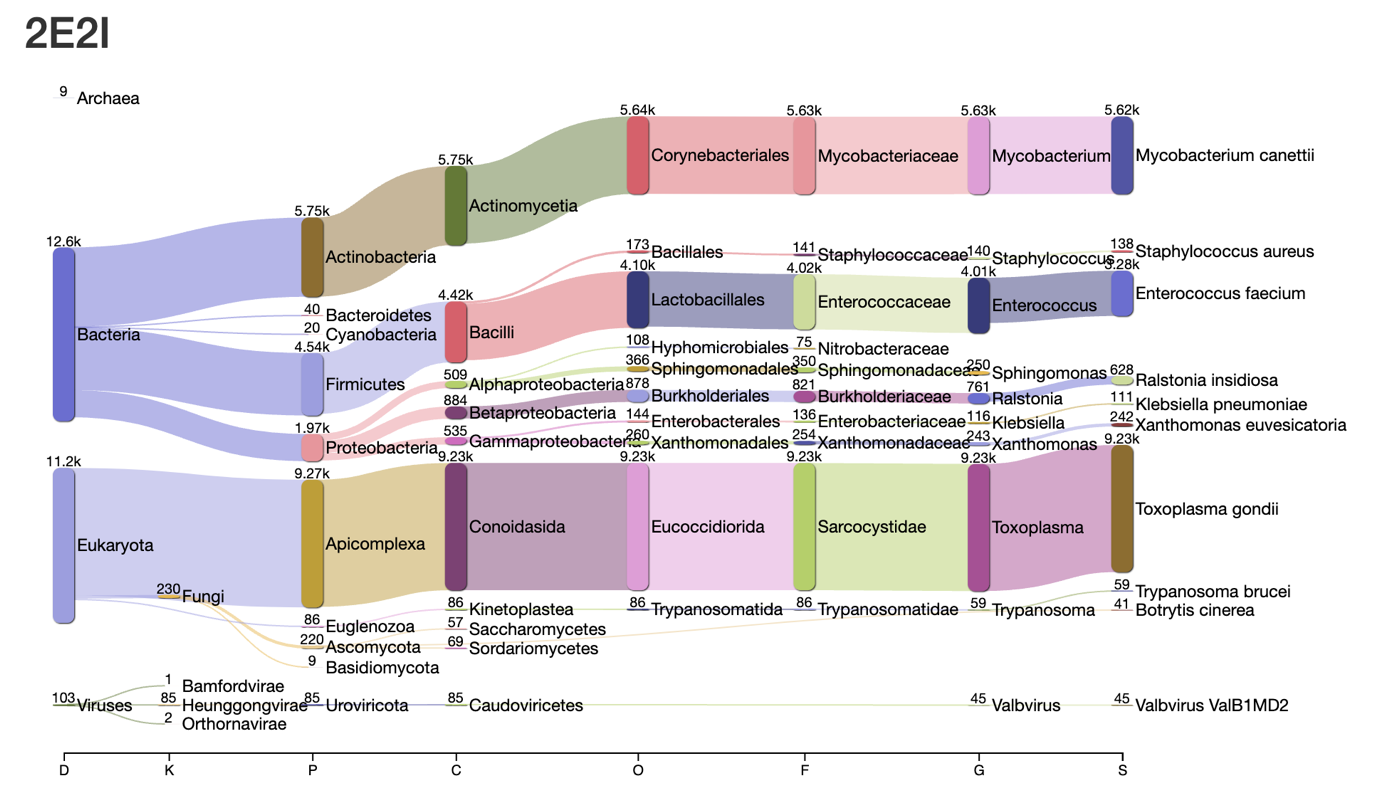


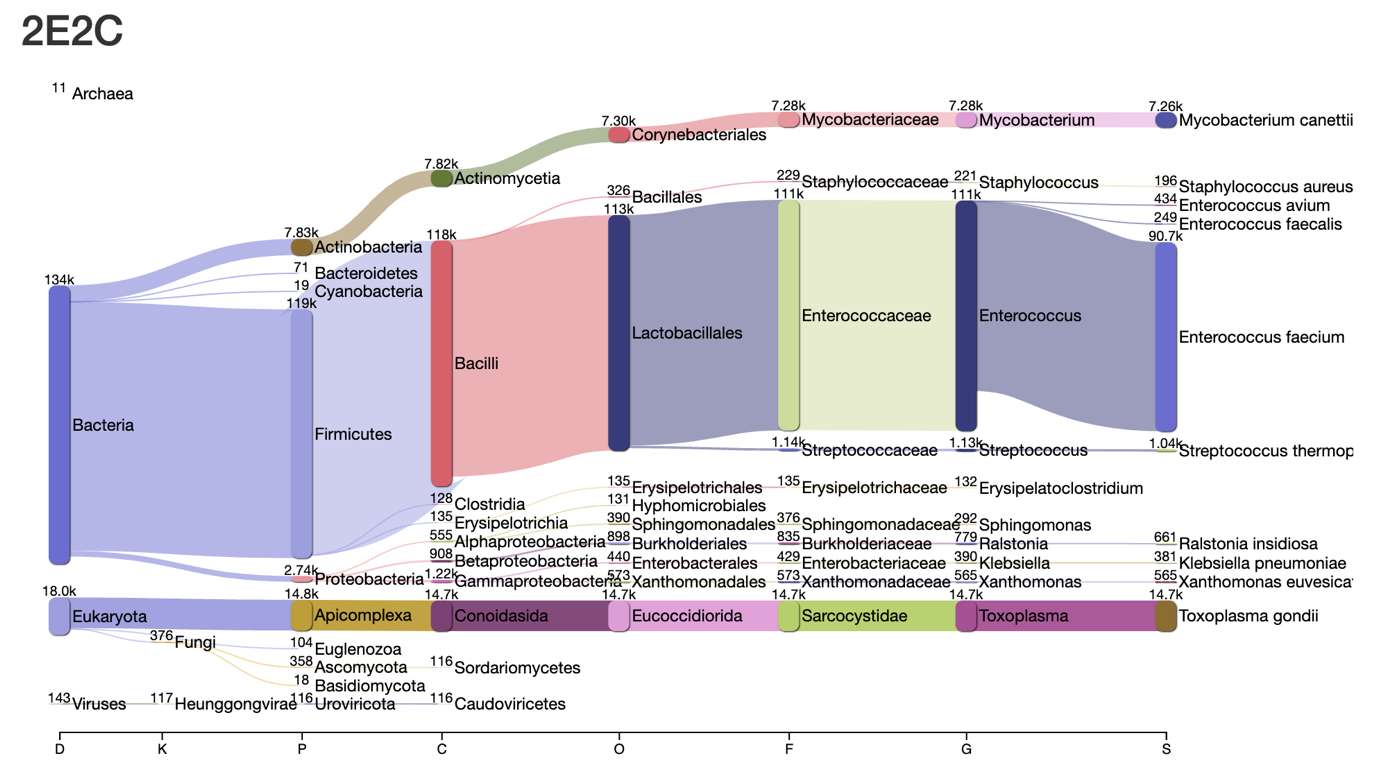


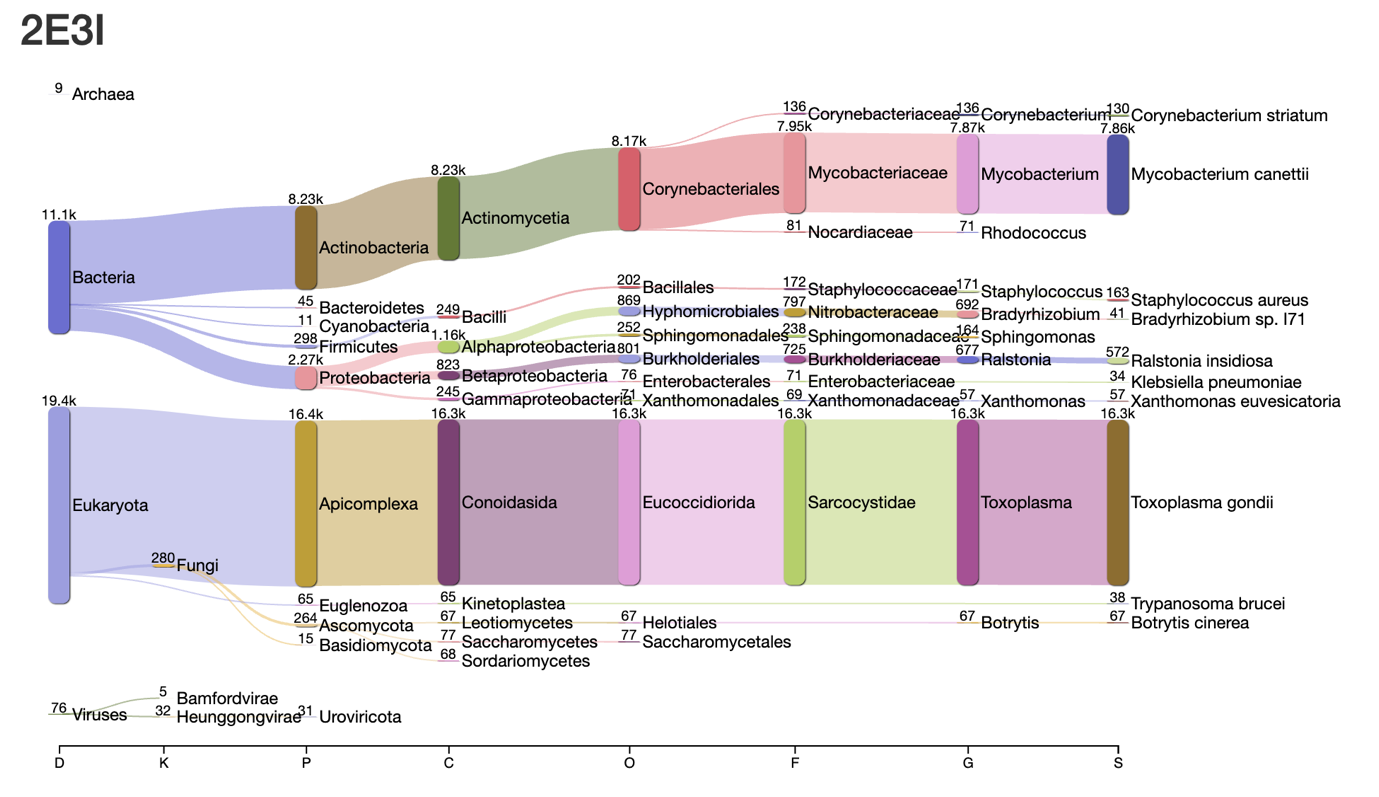


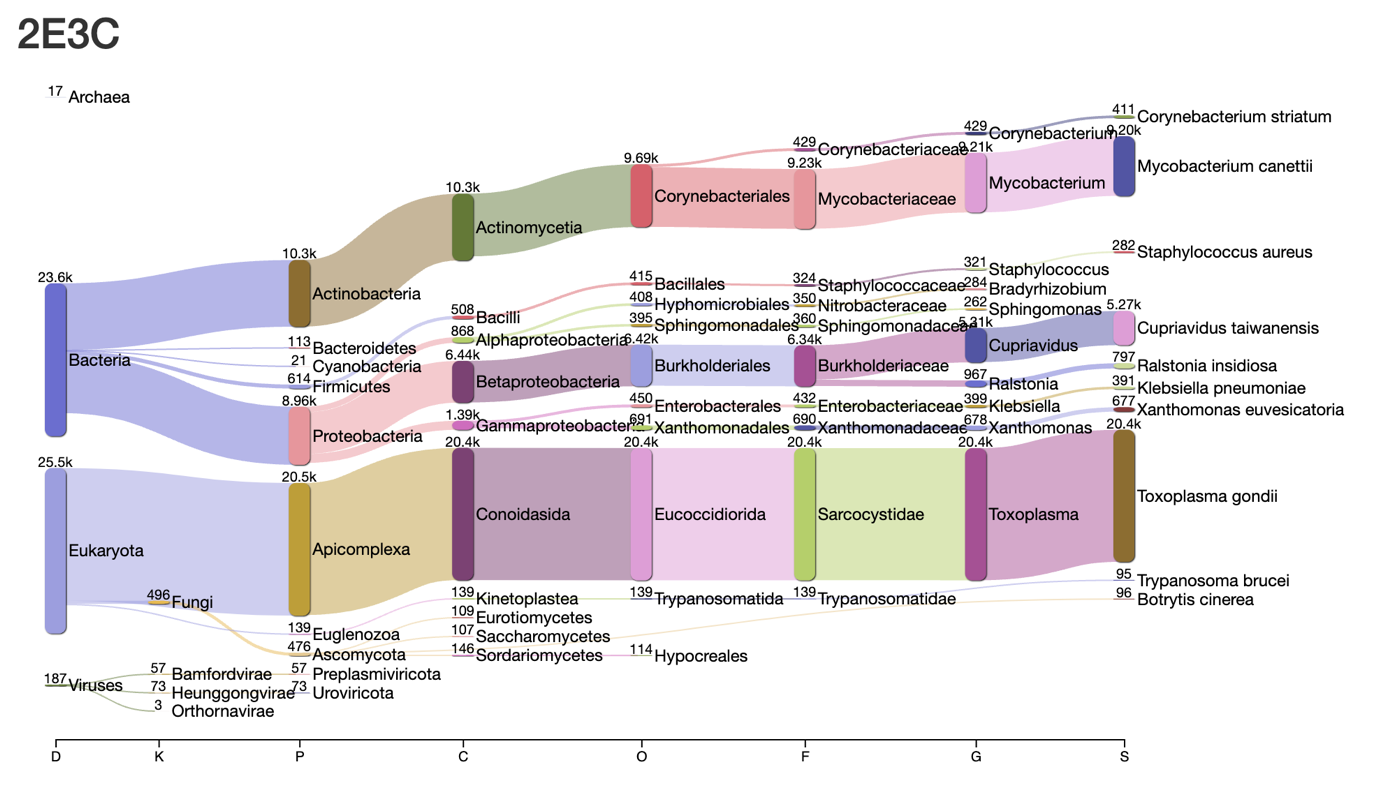


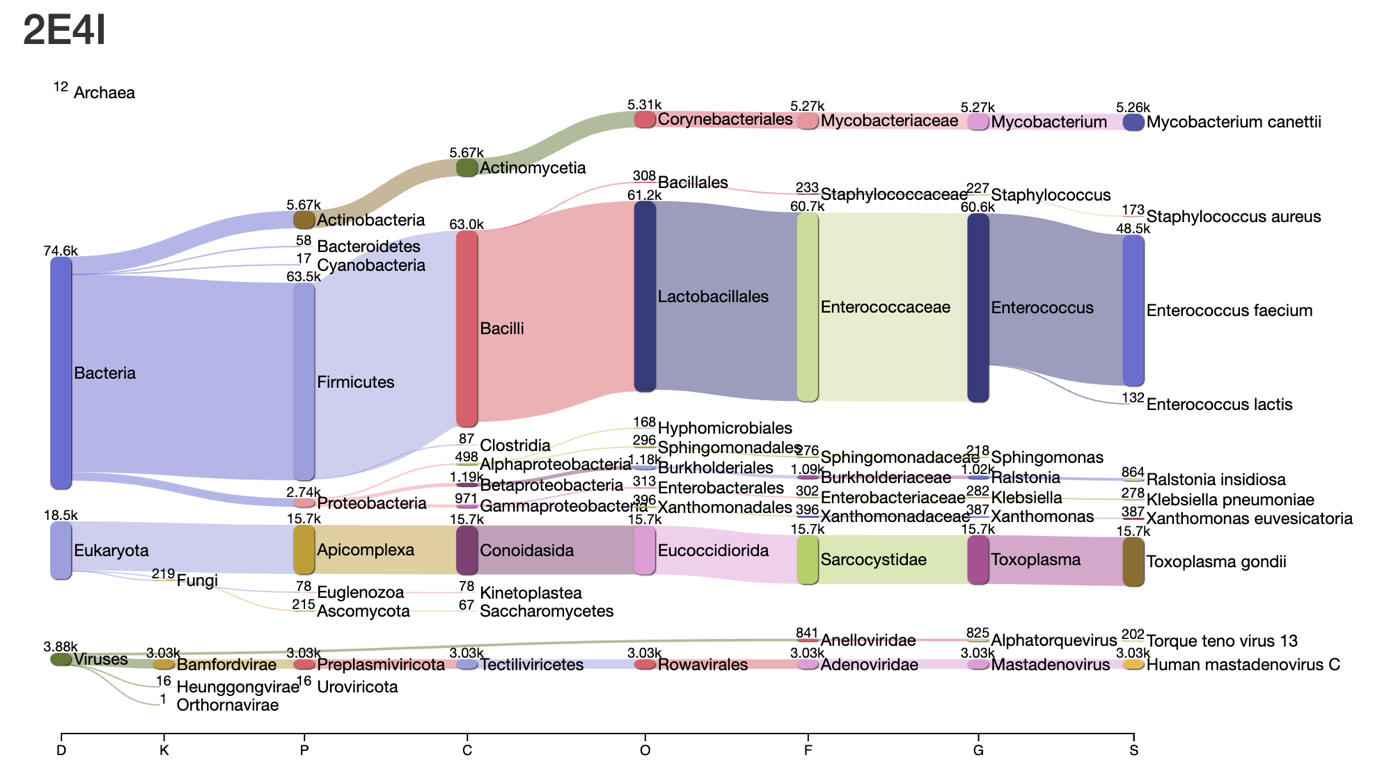


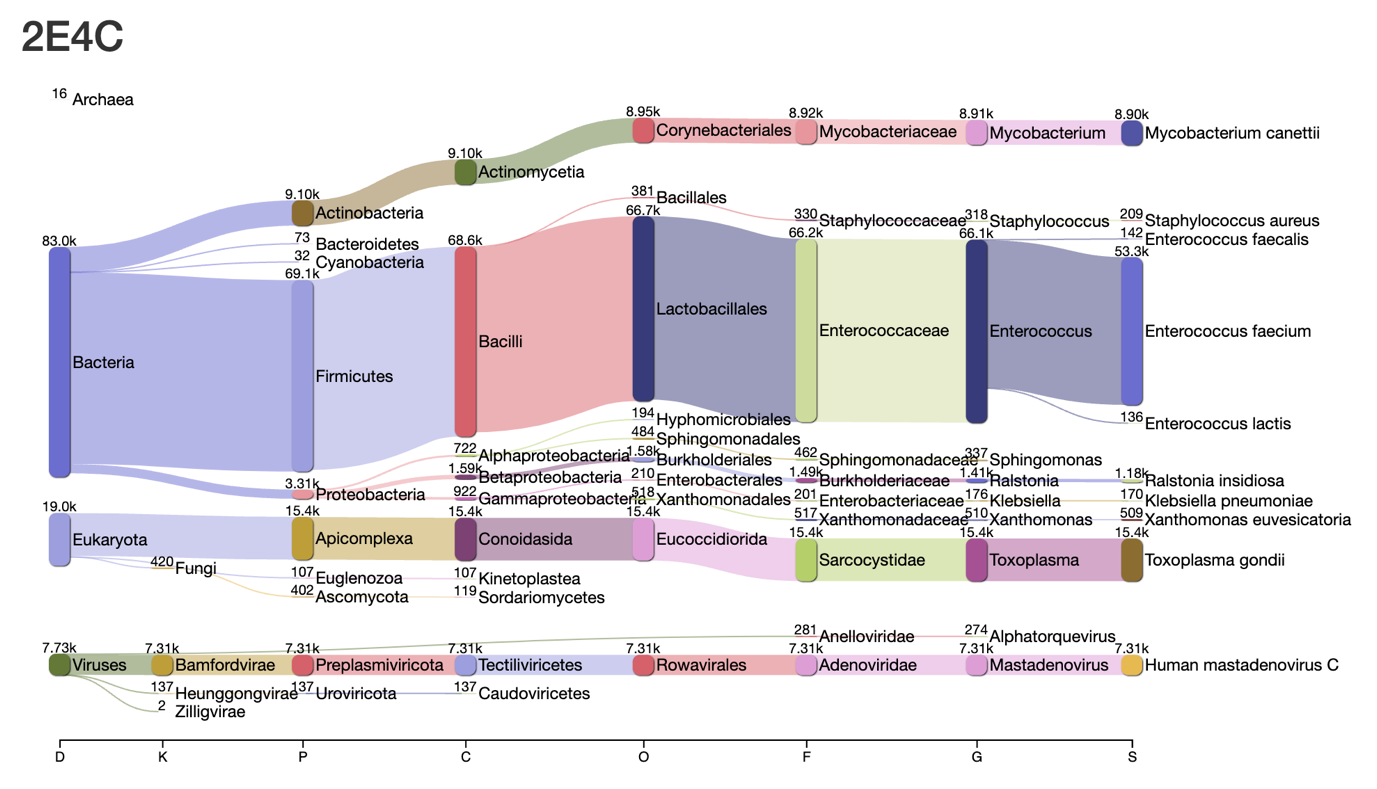


***Participant 3***


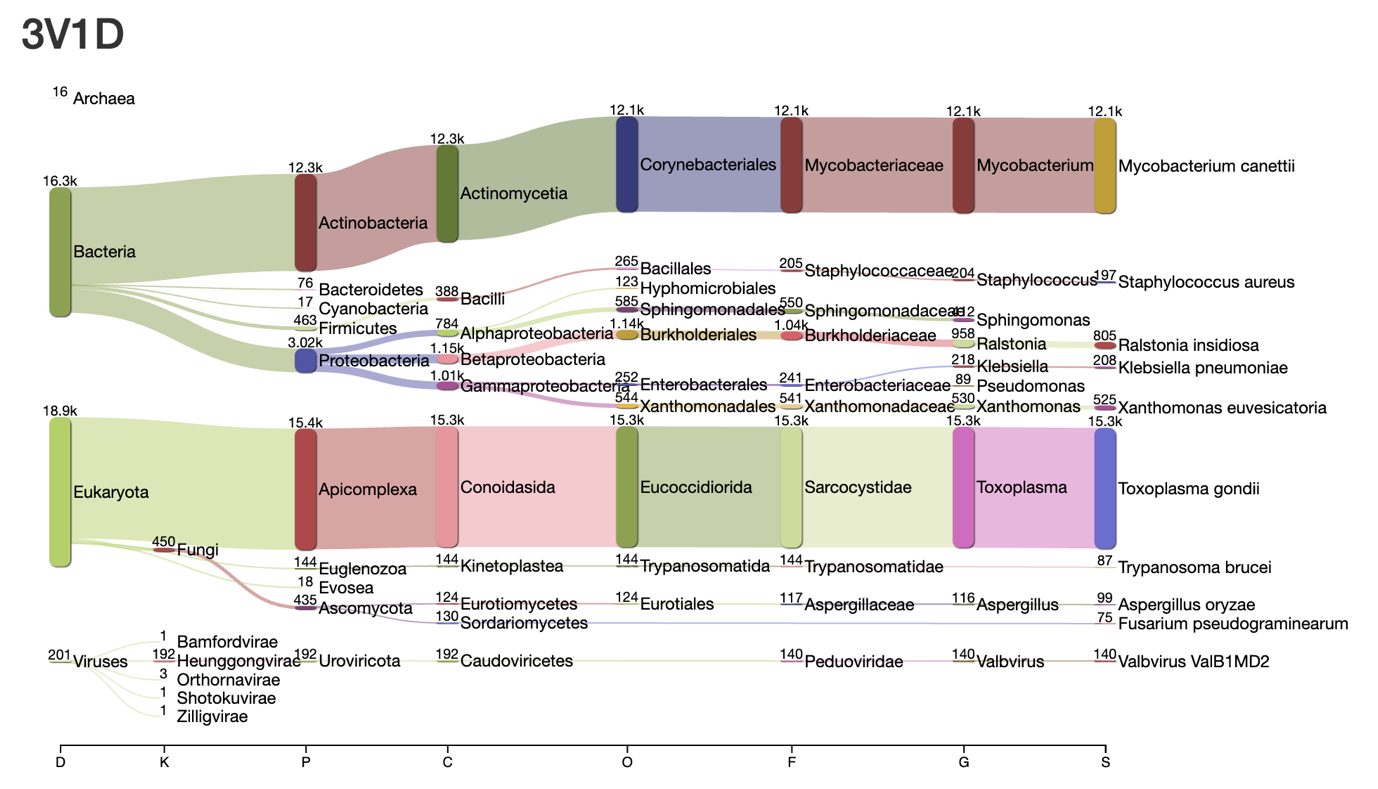


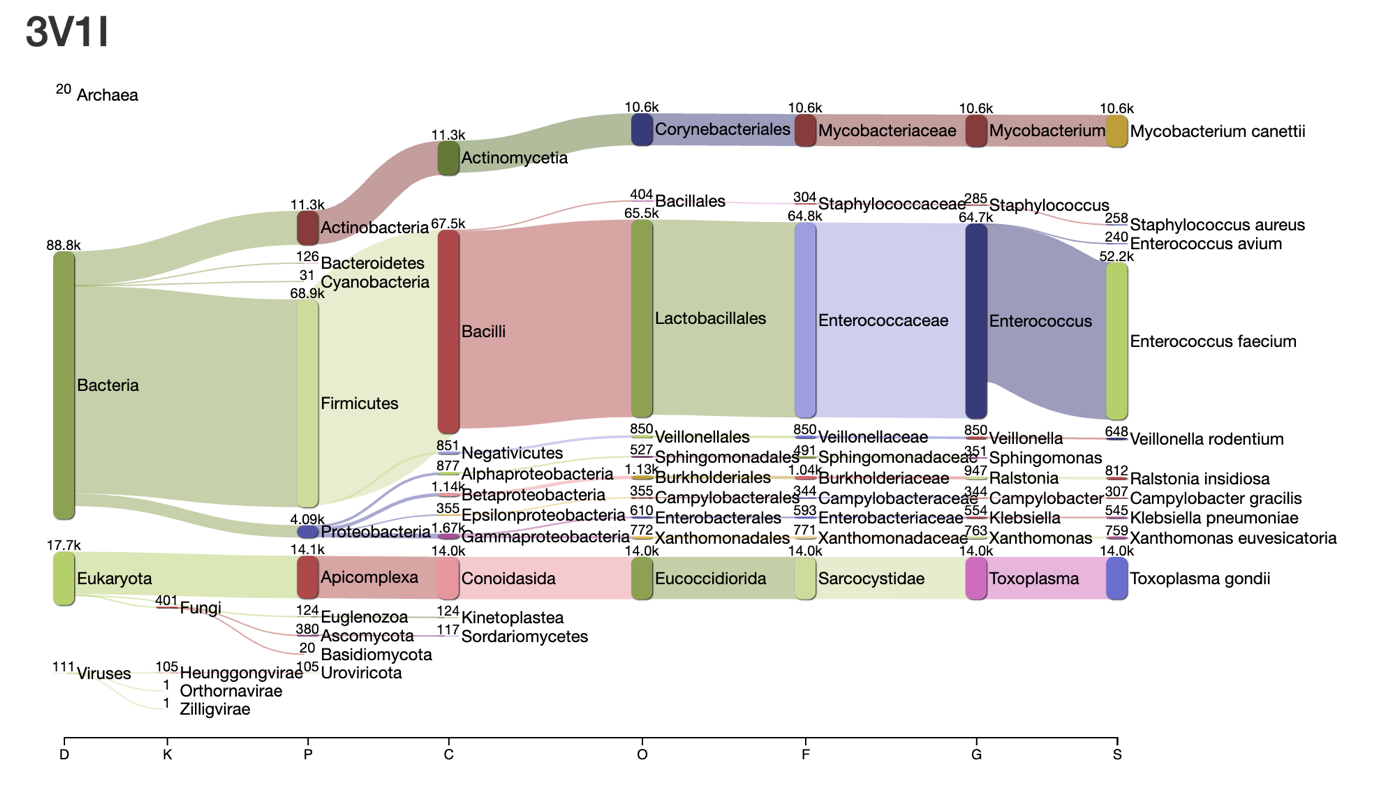


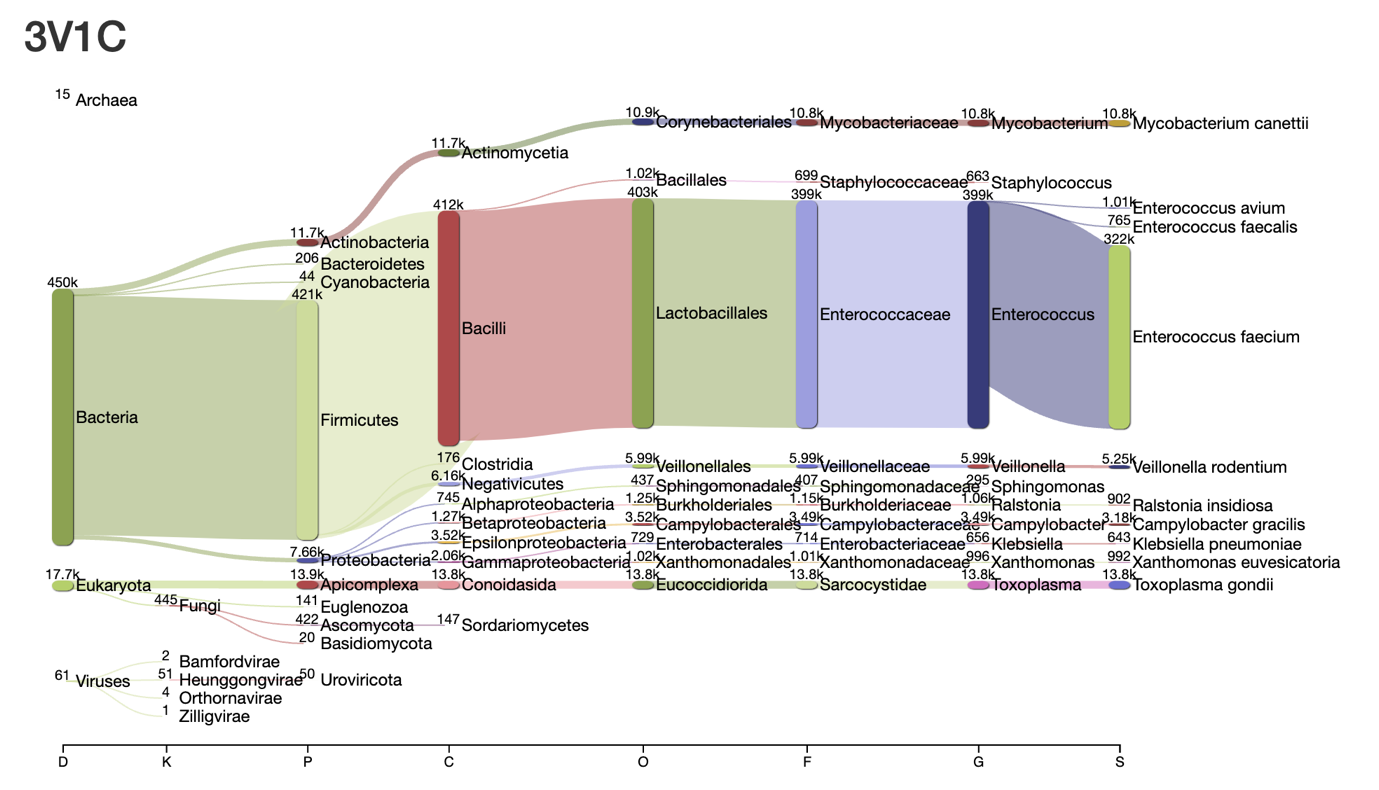


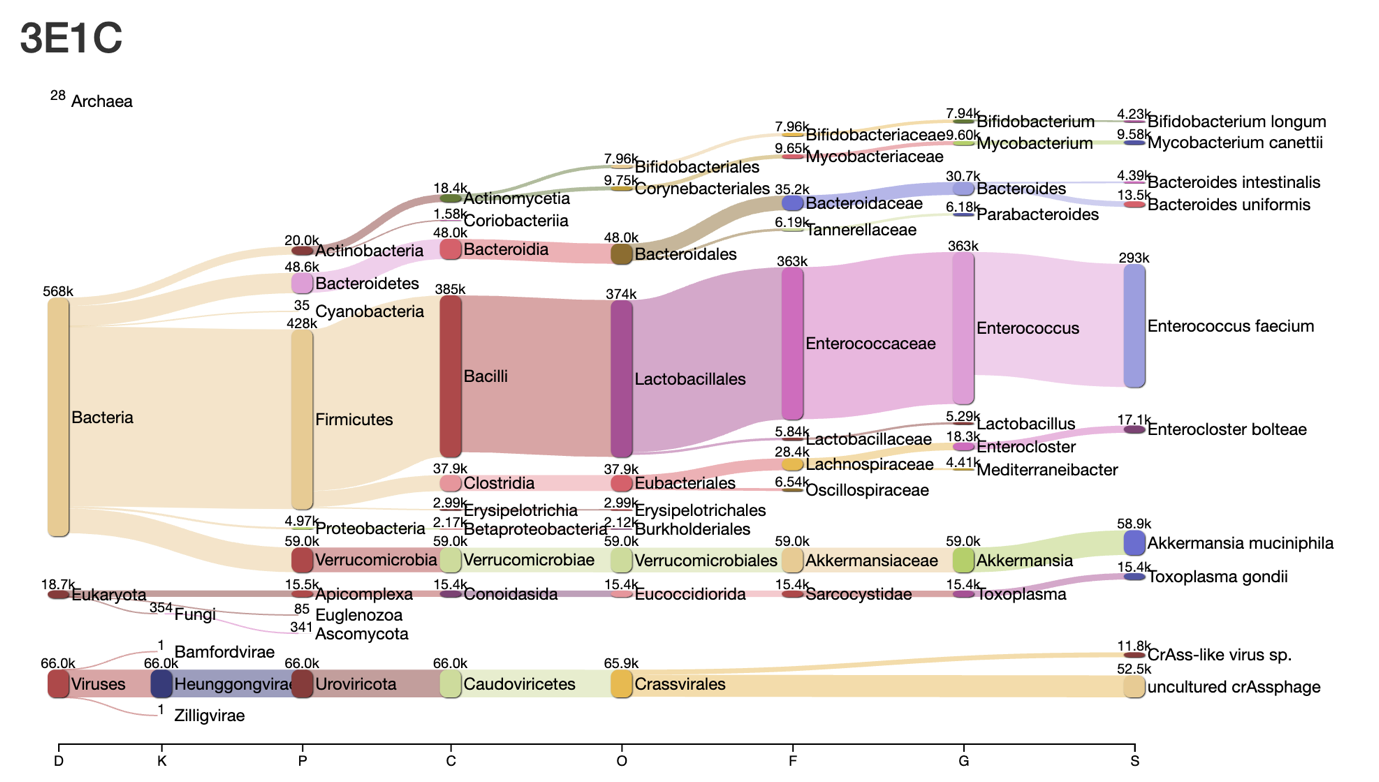


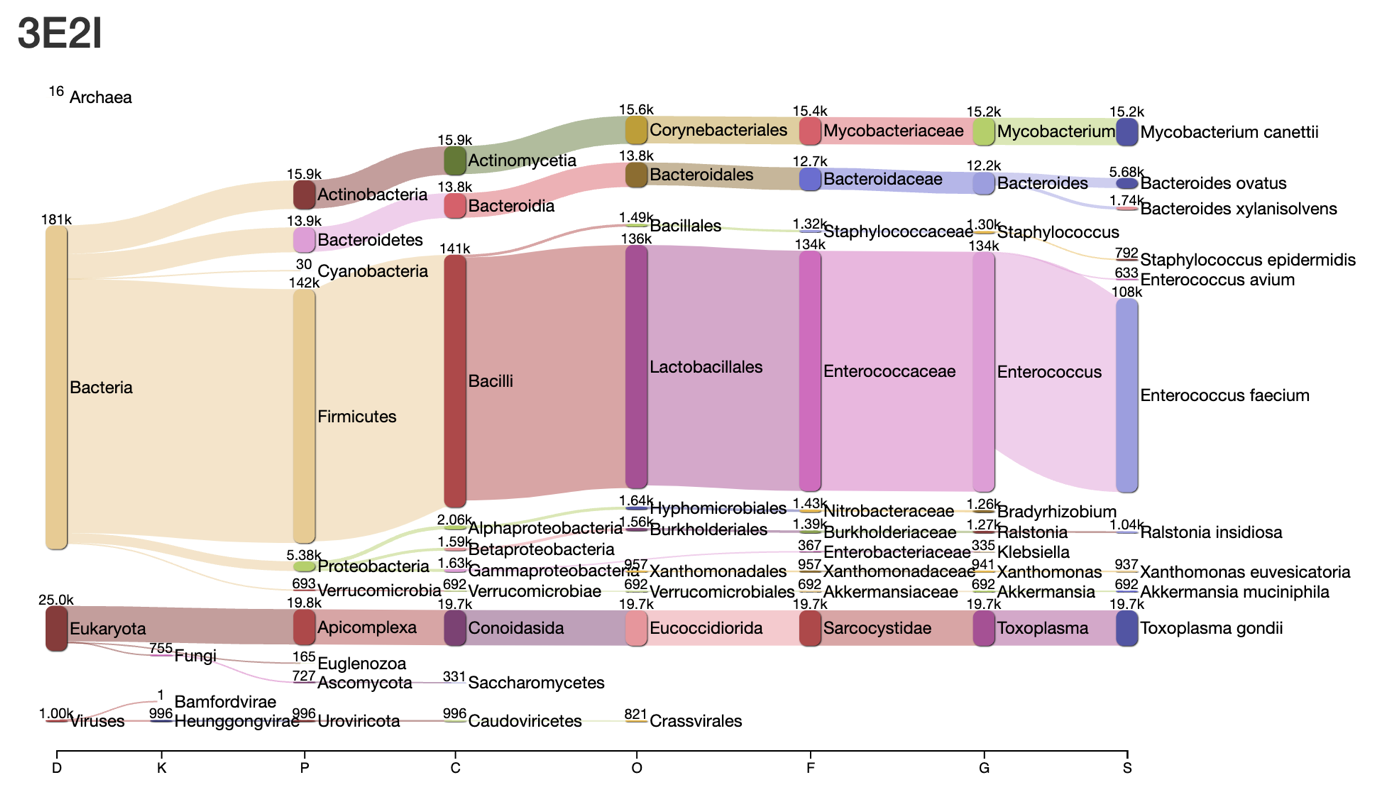


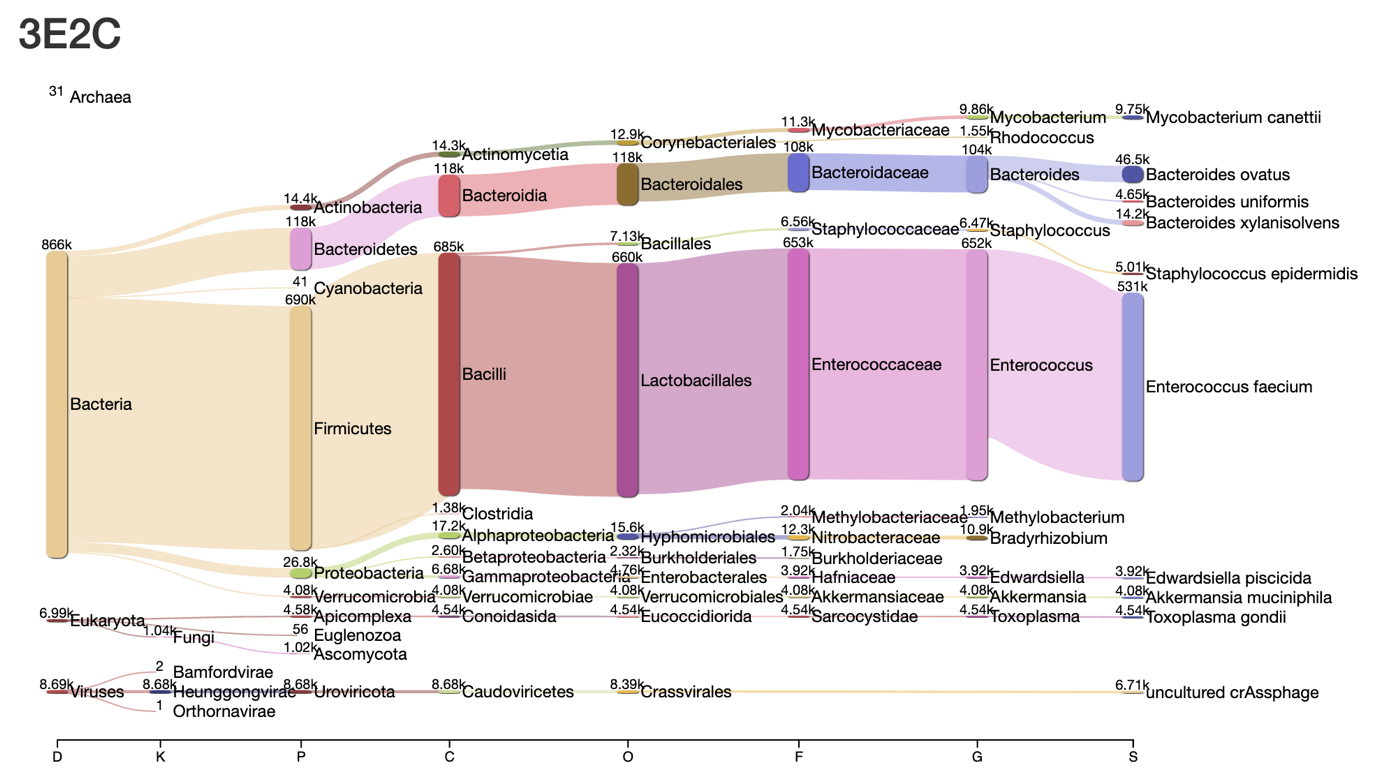


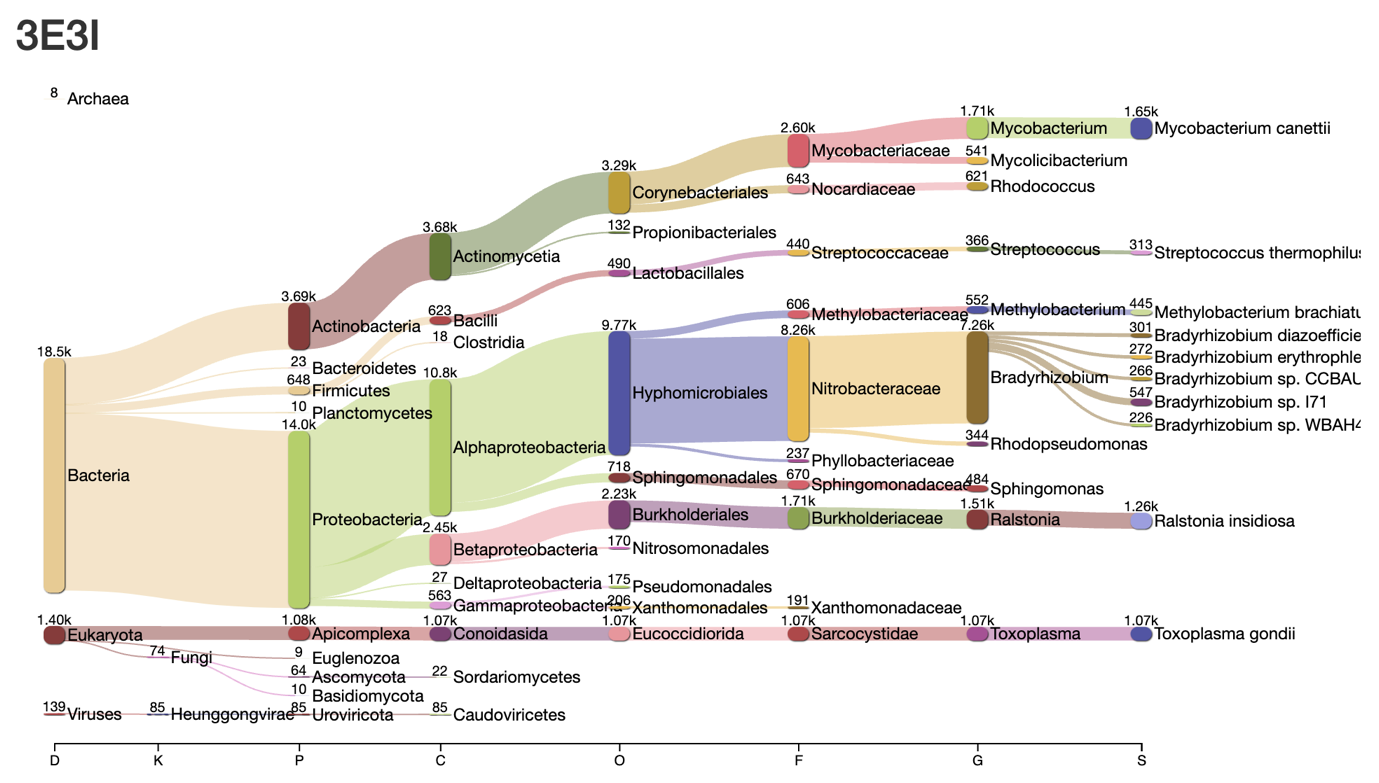


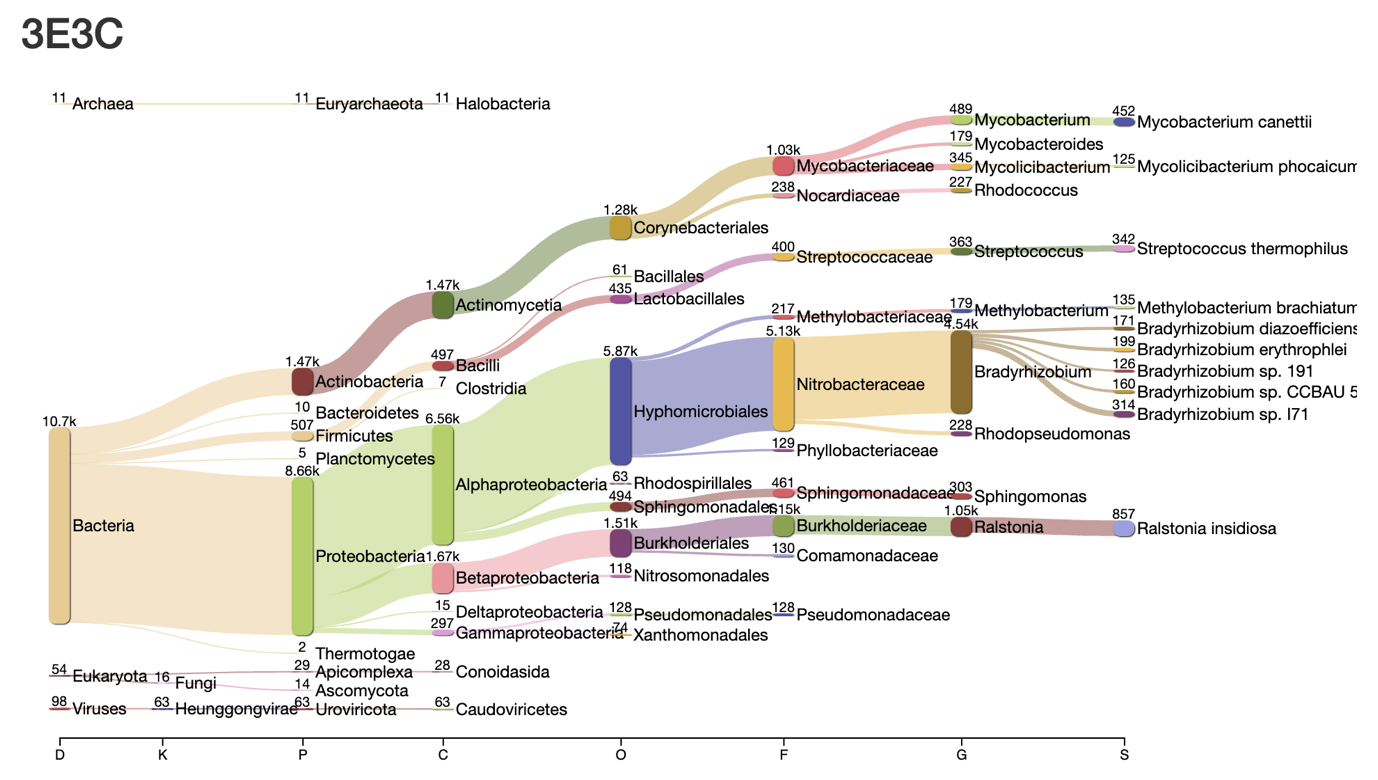


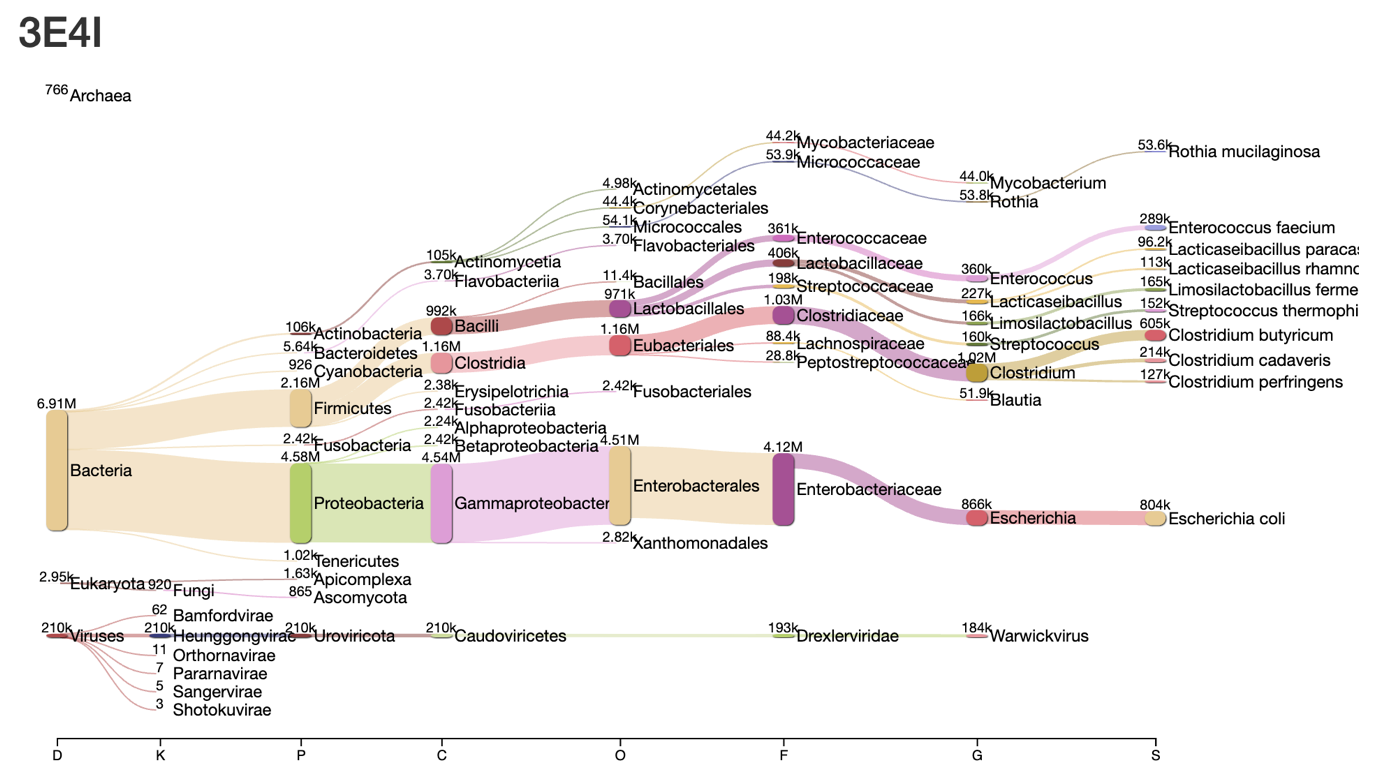


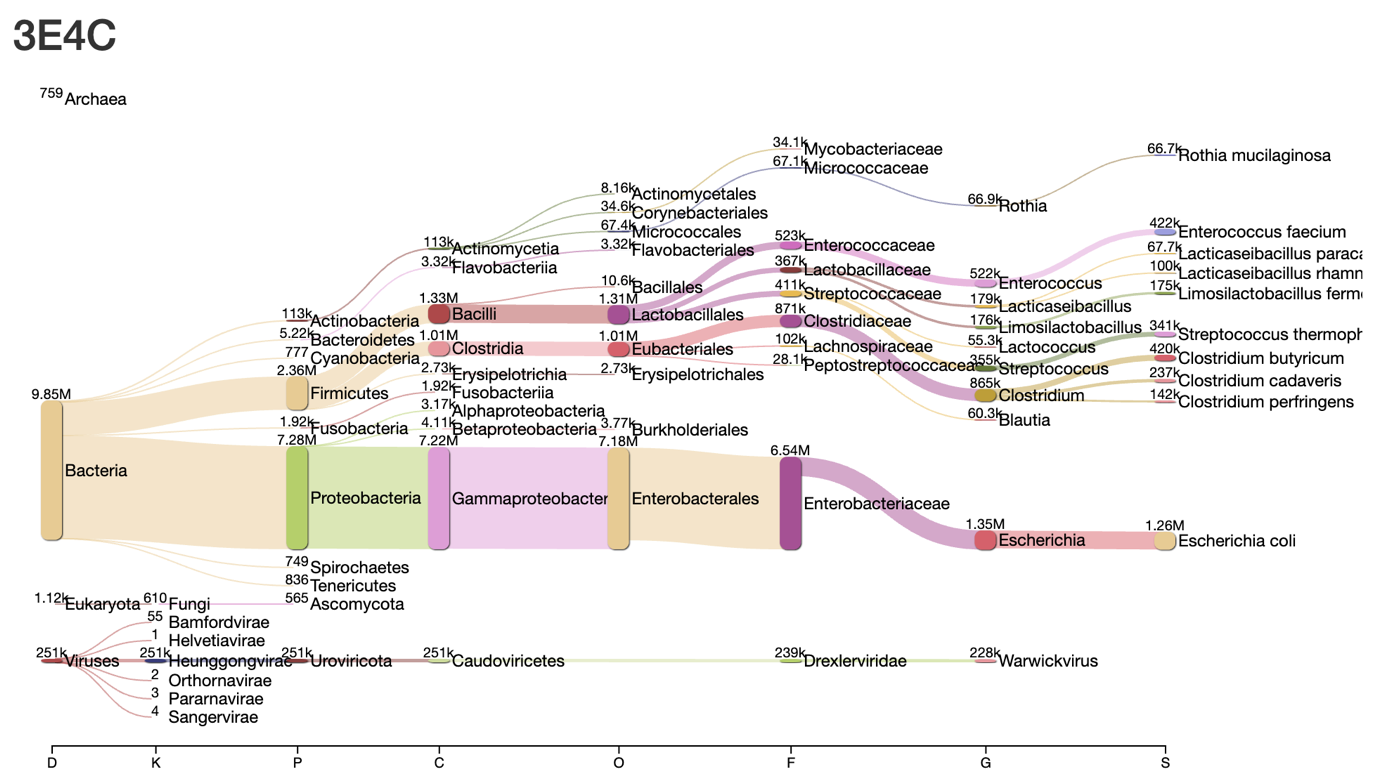


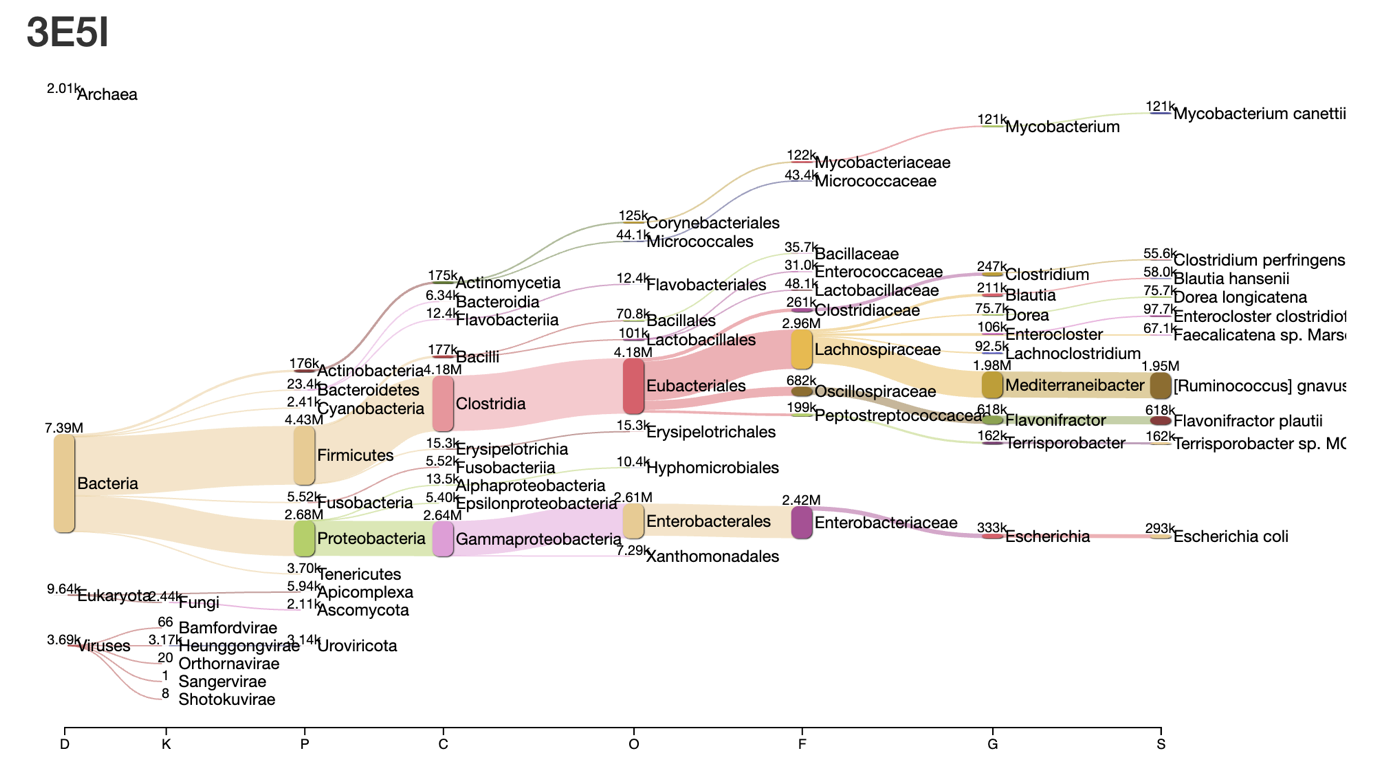


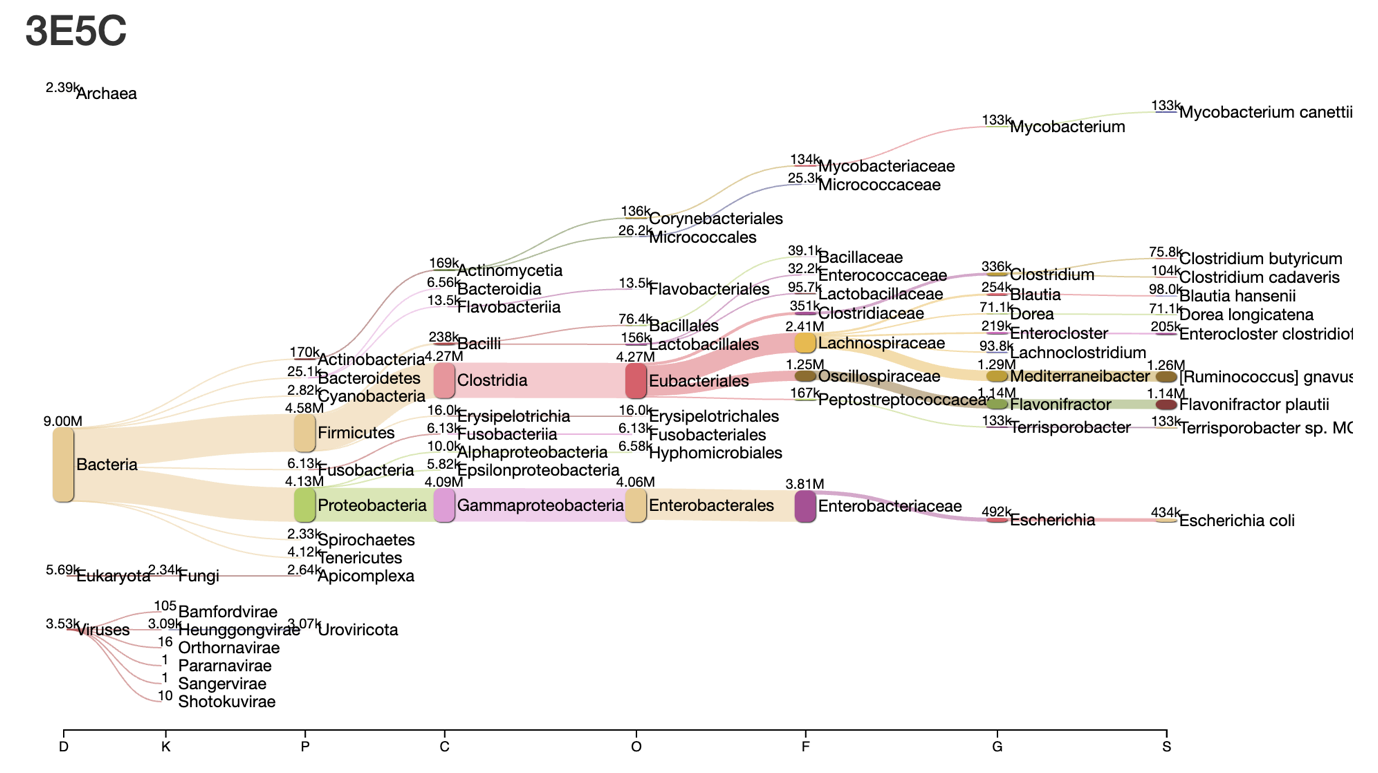


***Participant 4***


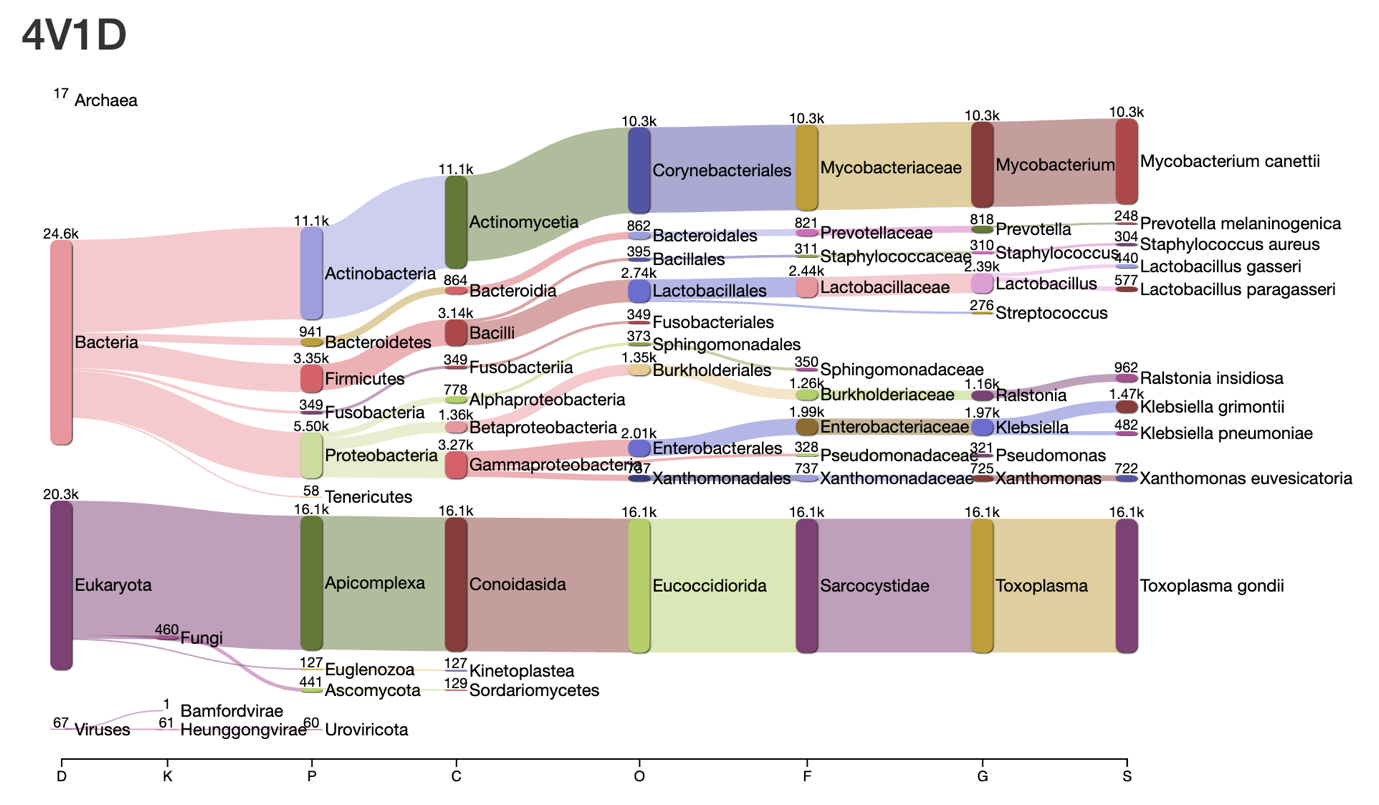


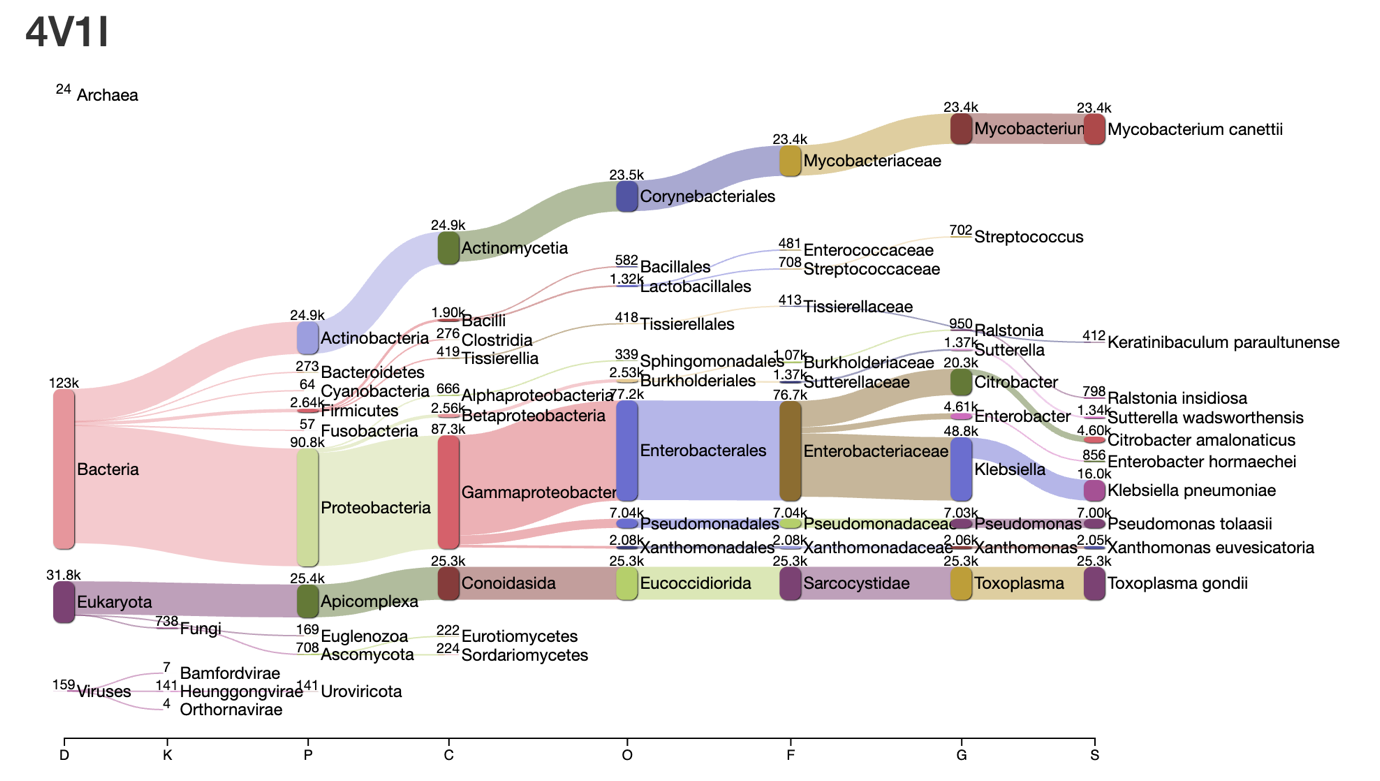

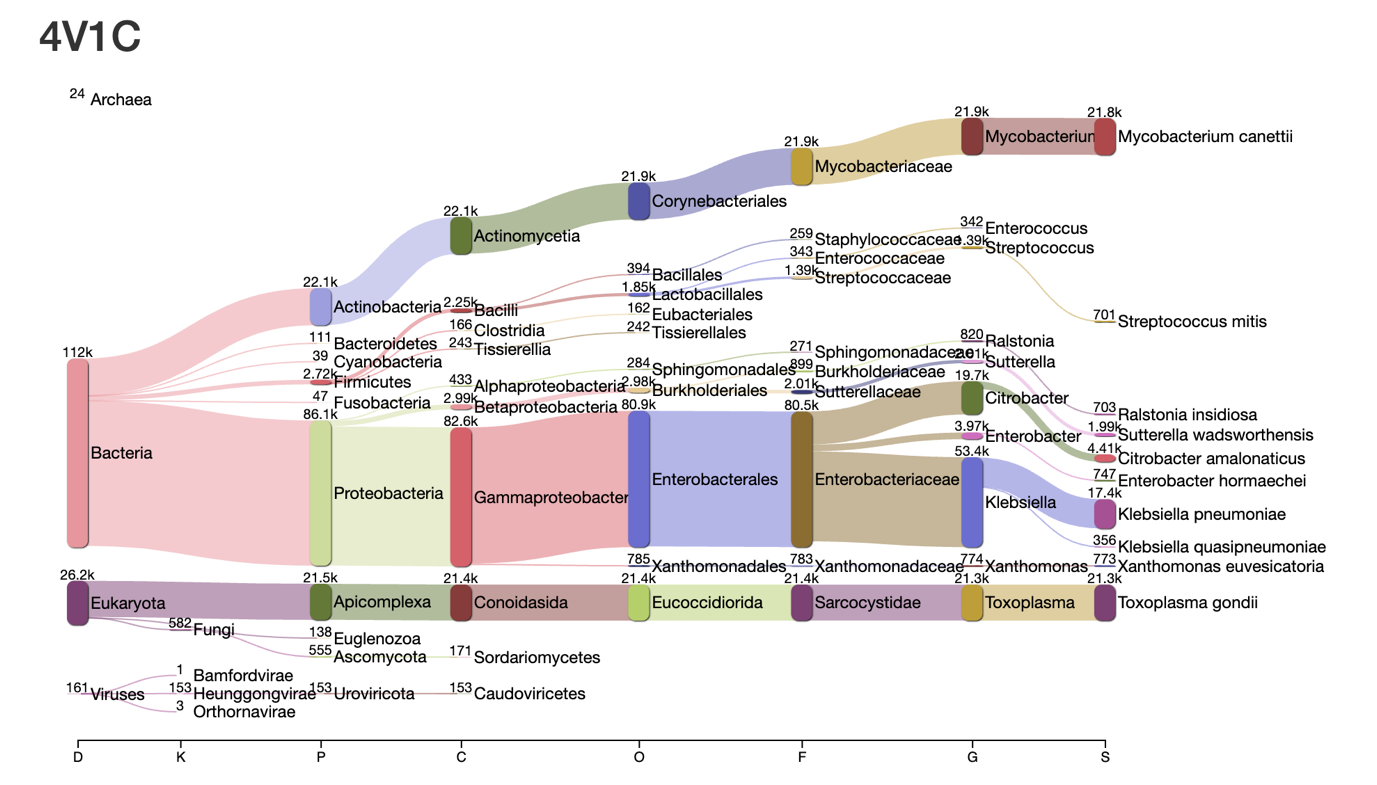


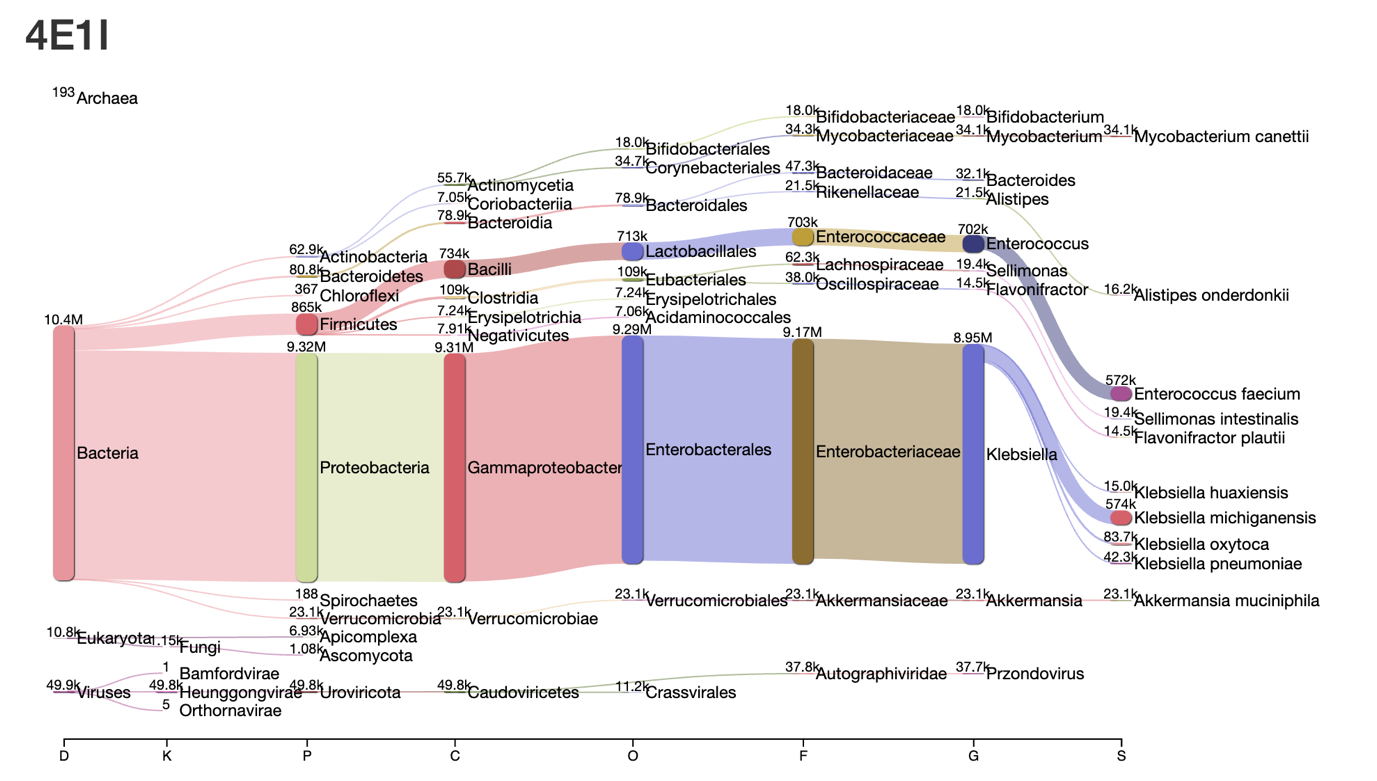

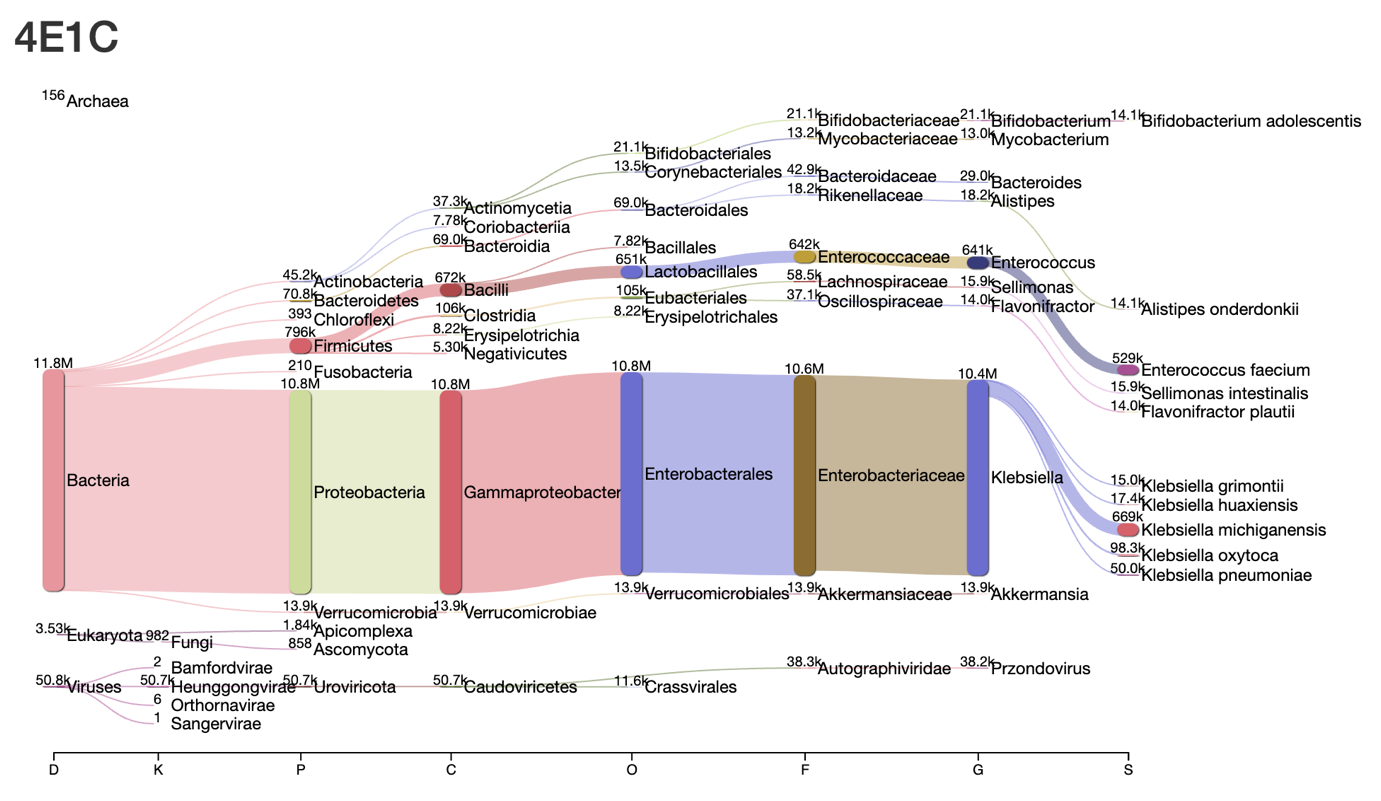


***Participant 5***


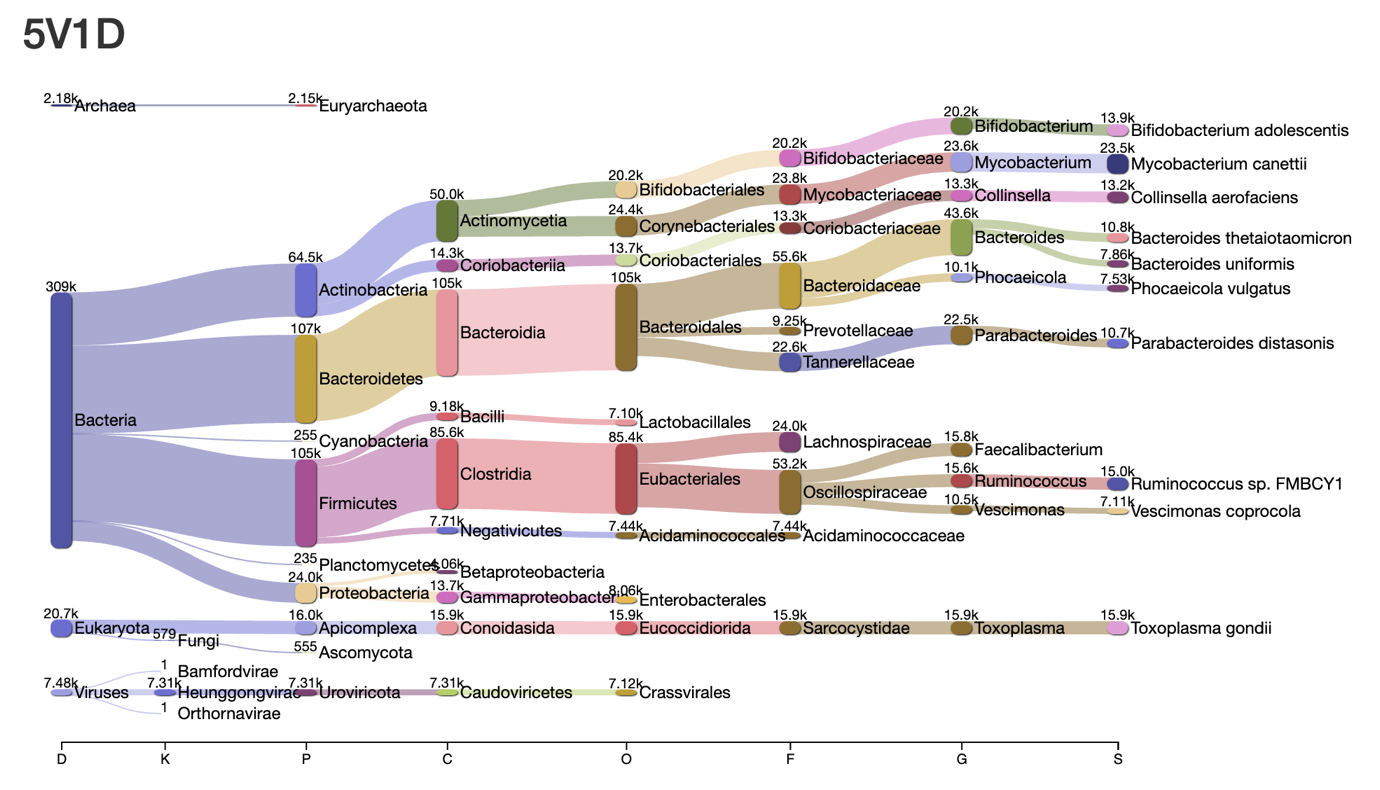


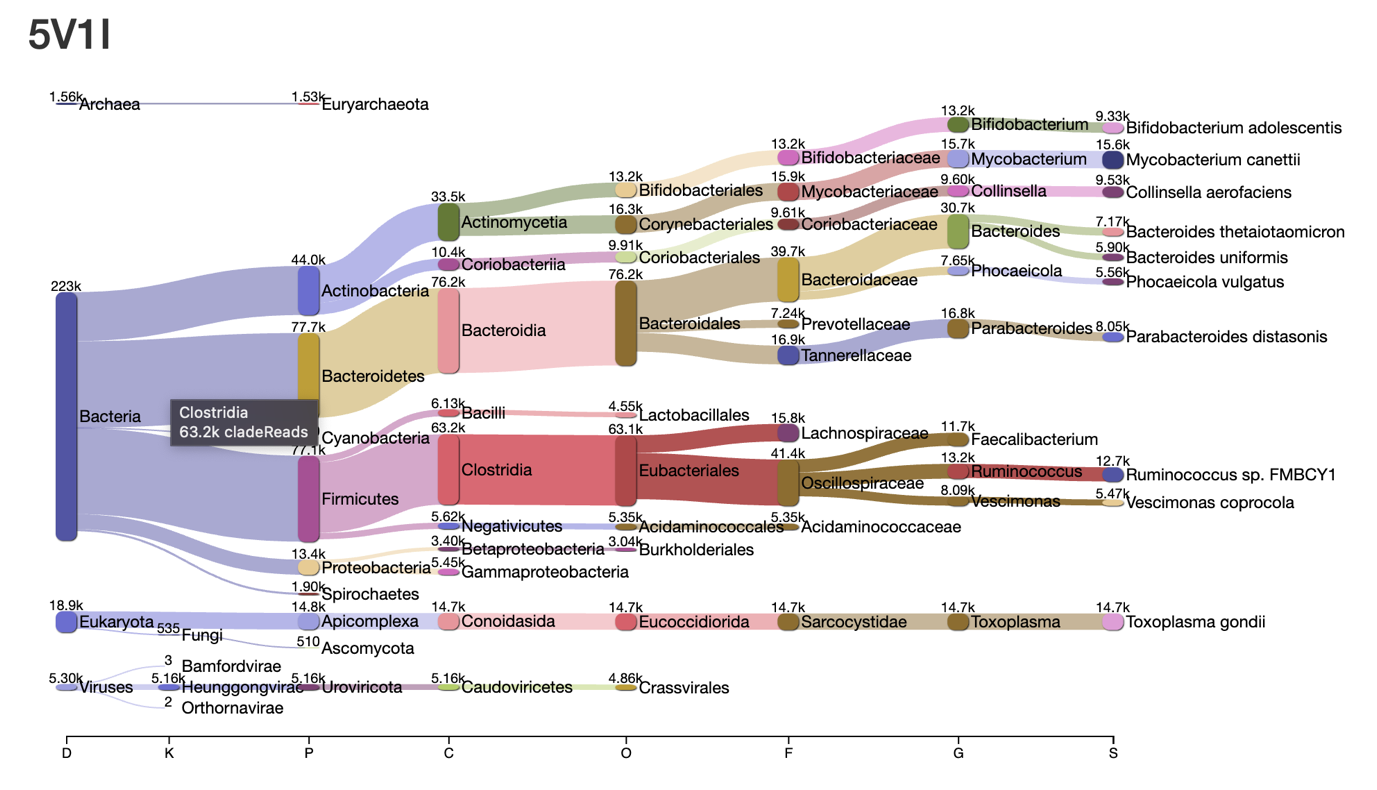

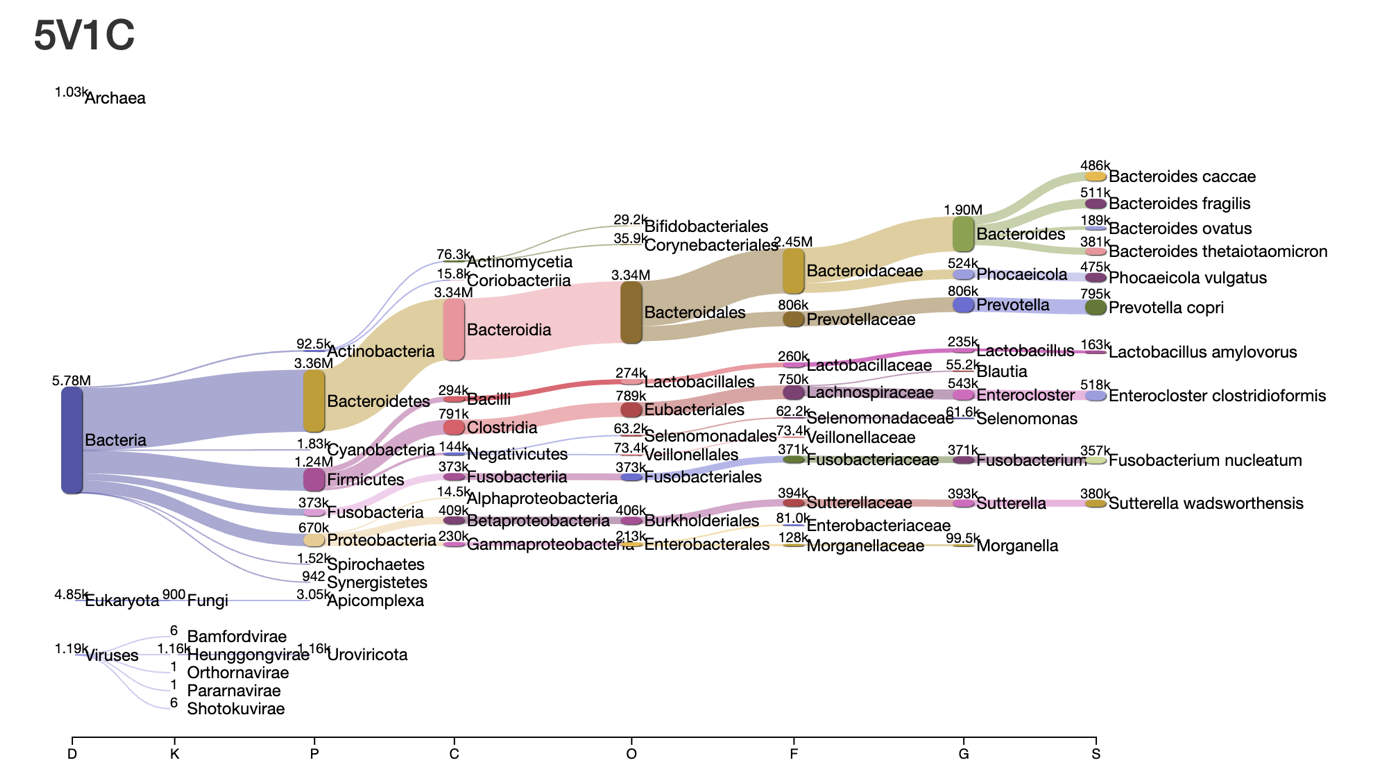

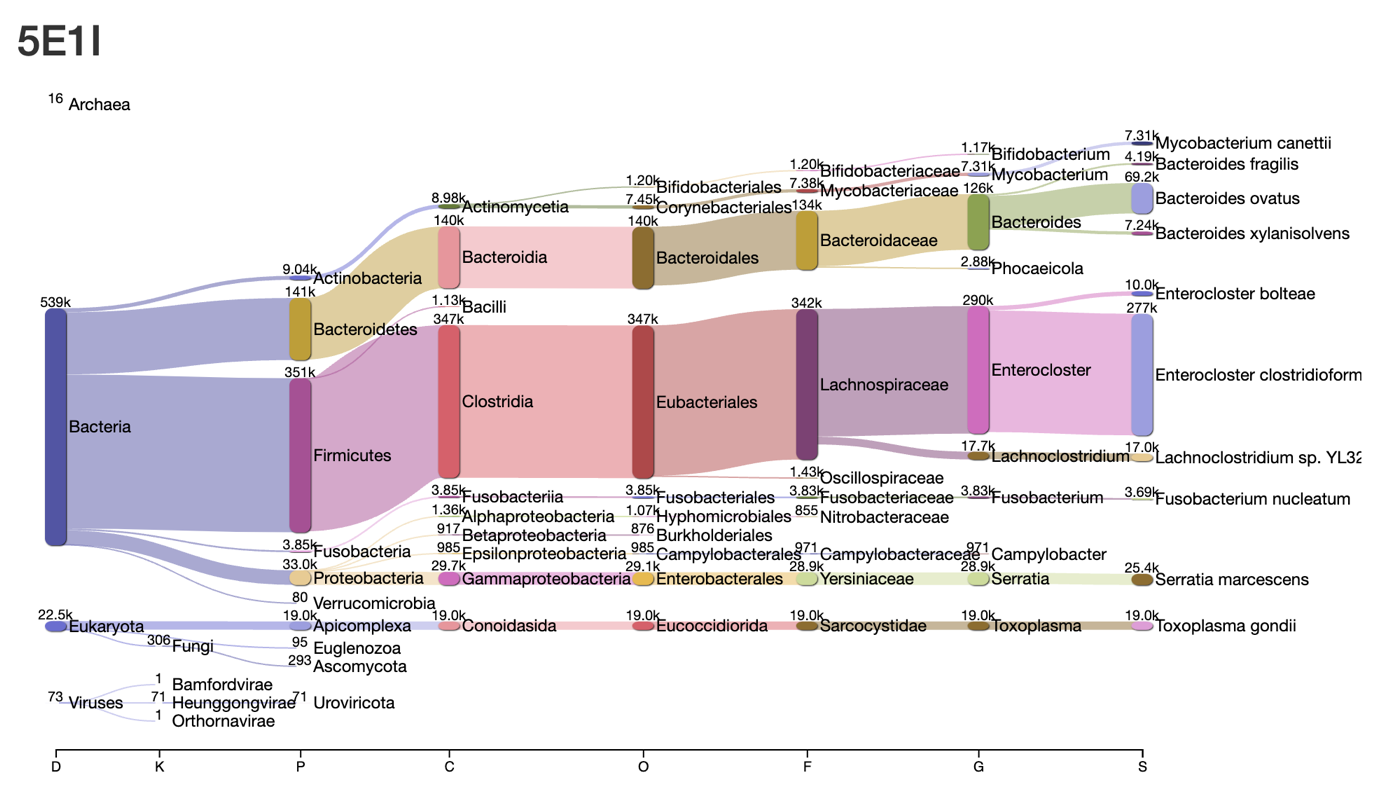


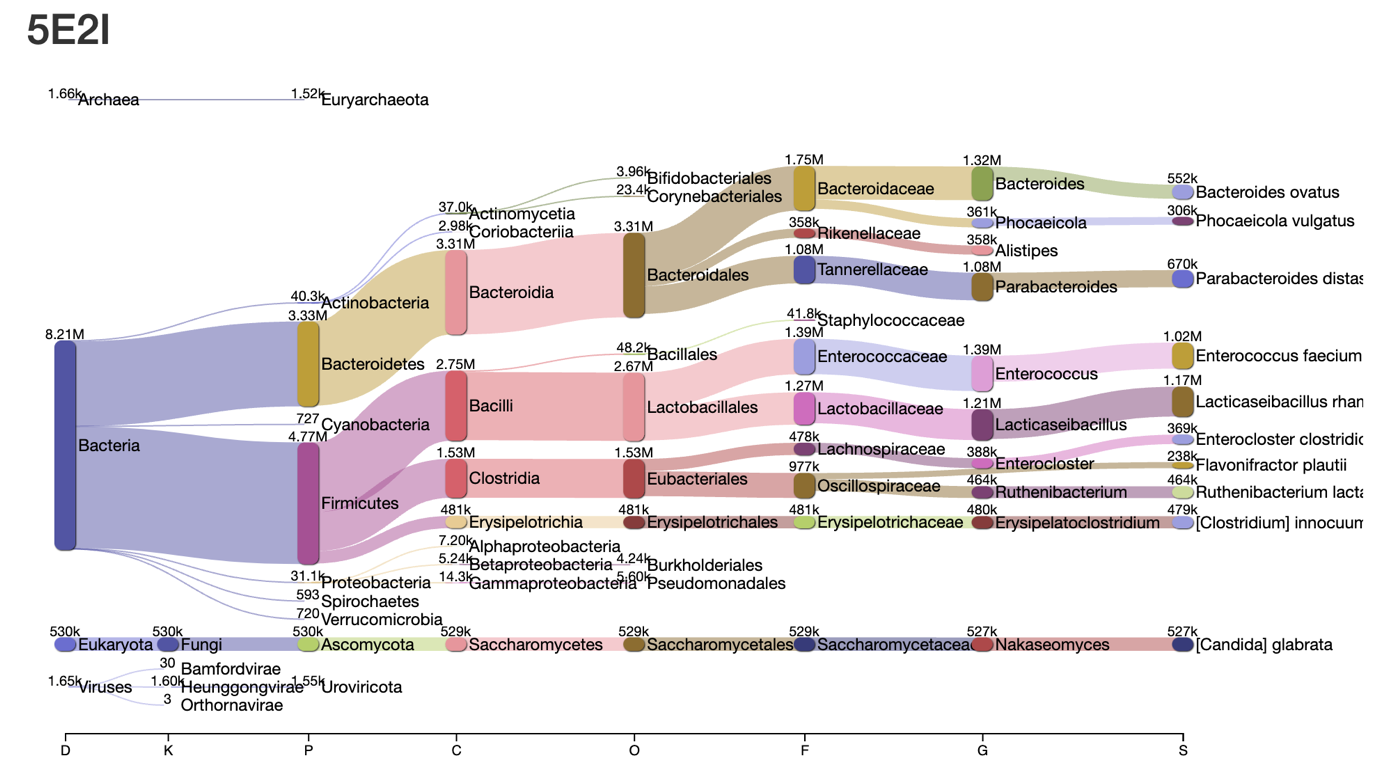

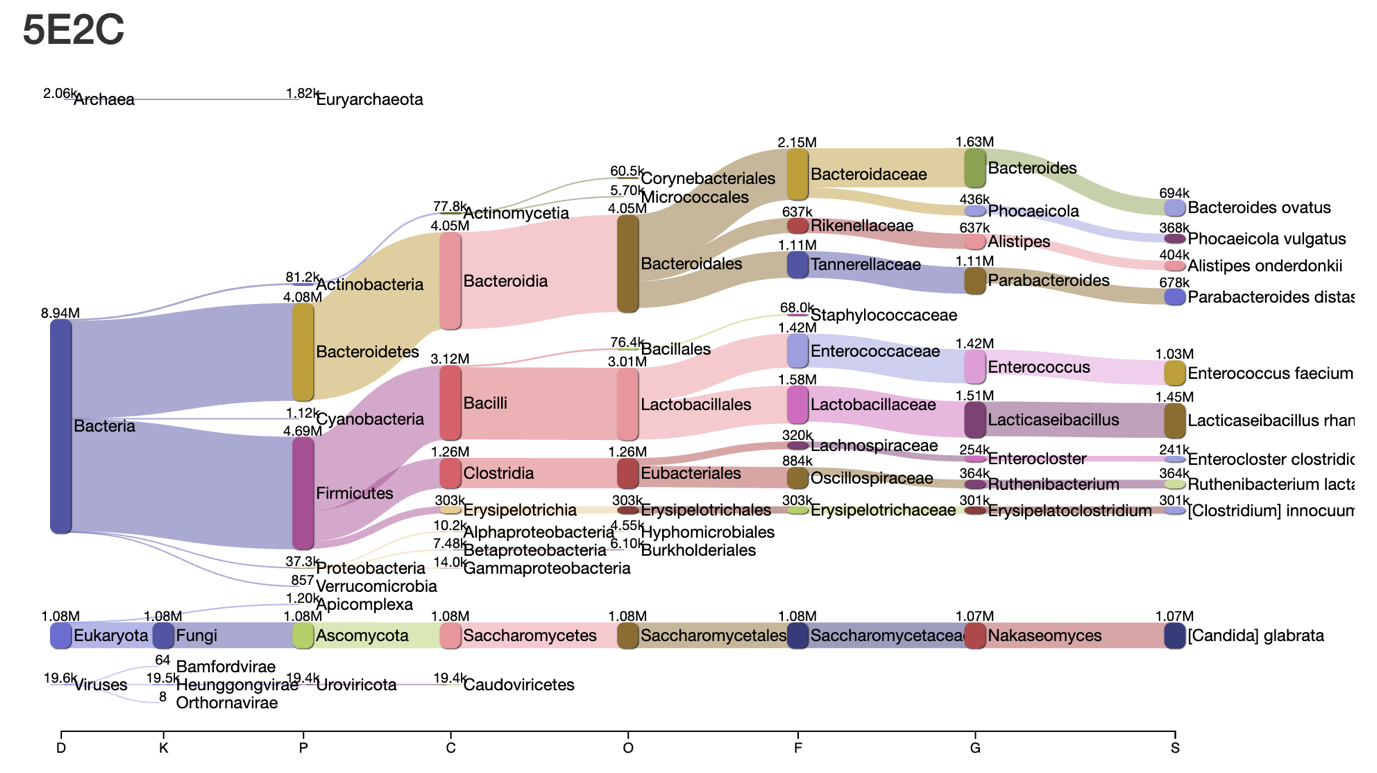


**Supplementary figure 2: InStrain dendrograms depicting shared genome coverage and ANI between gut microbiome samples in relation to sequenced clinical isolates from patient 2 (002VRE), patient 3 (003VRE) and patient 4 (004KOX).**

**
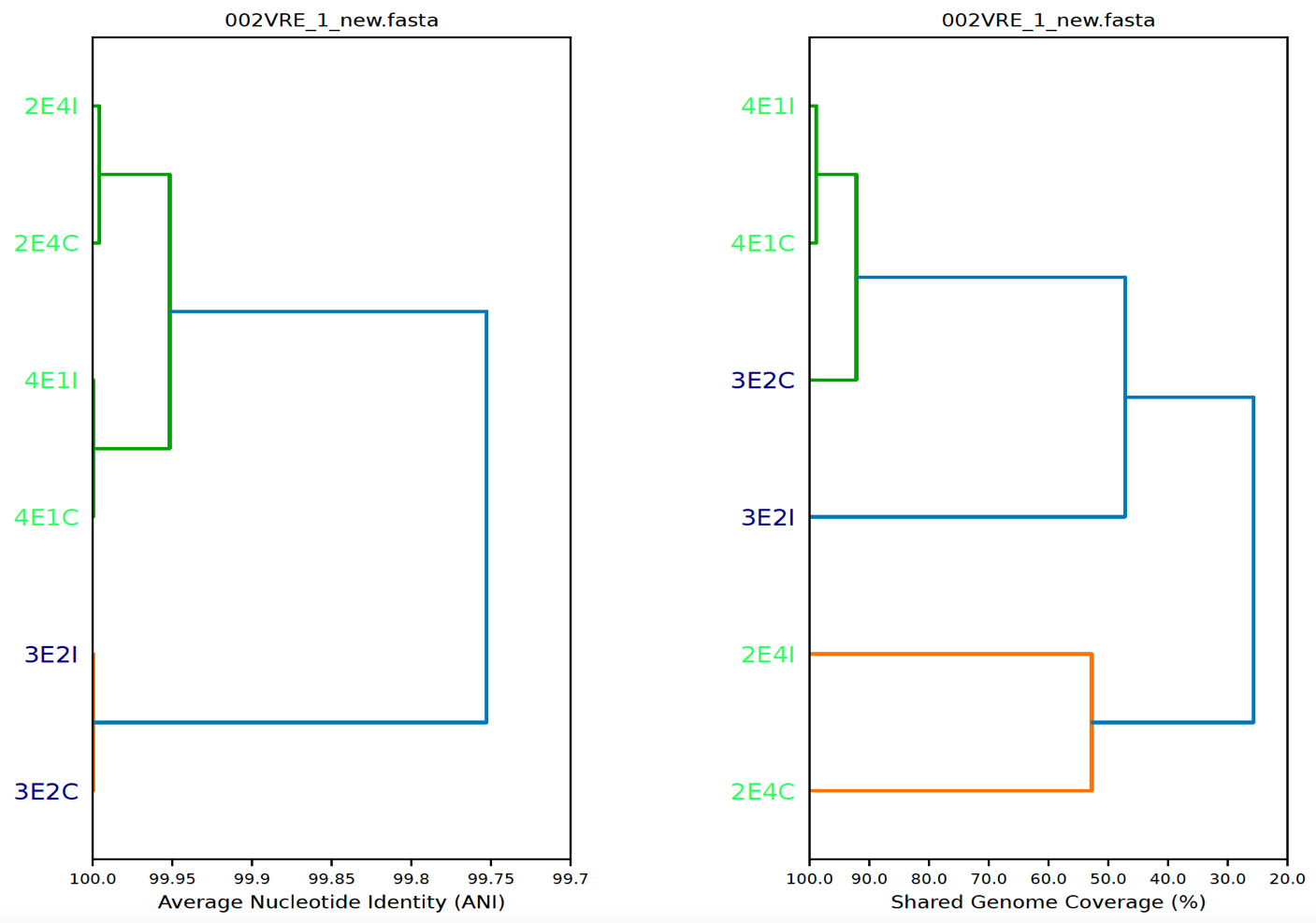
**

**
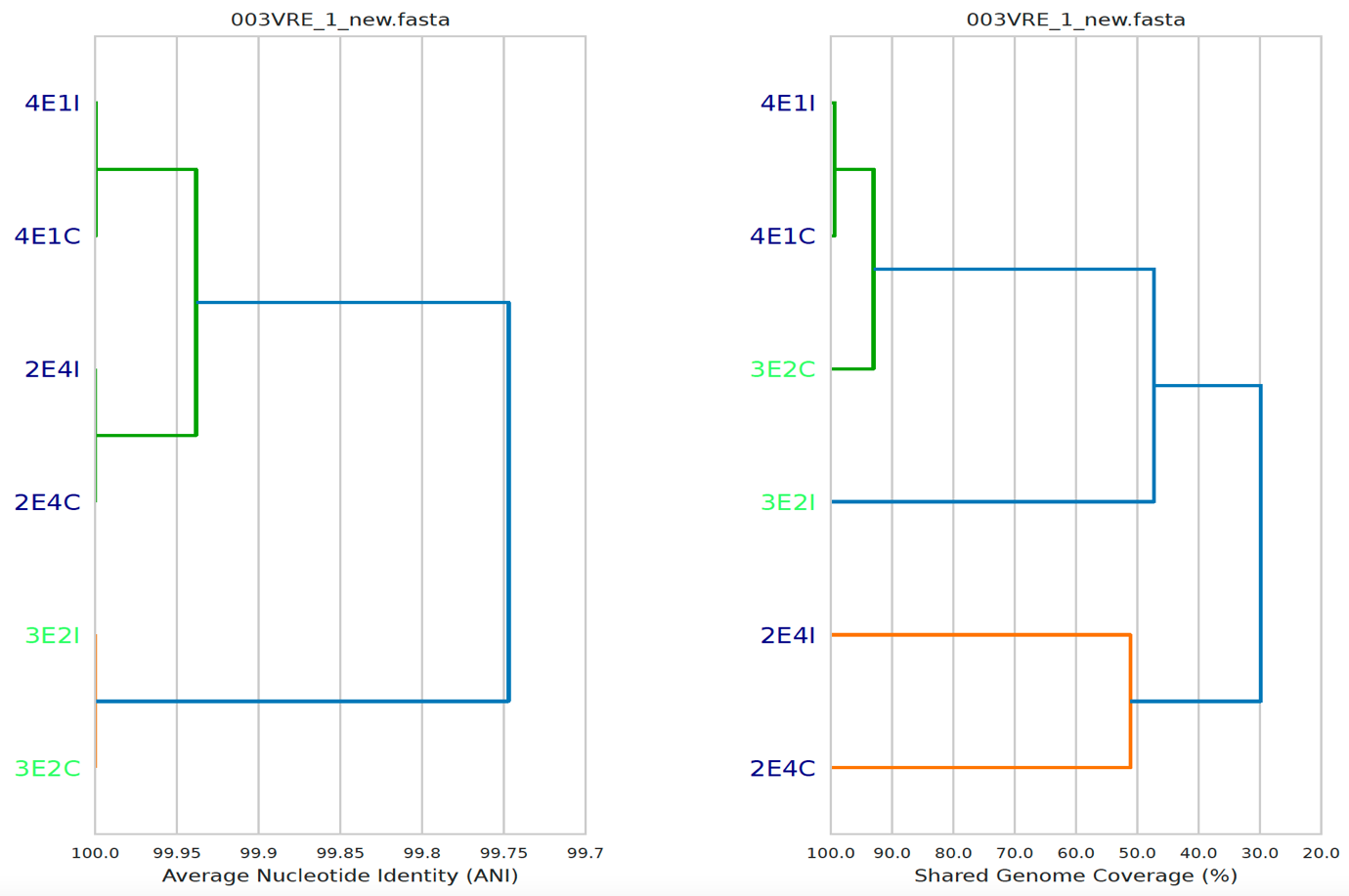
**

**
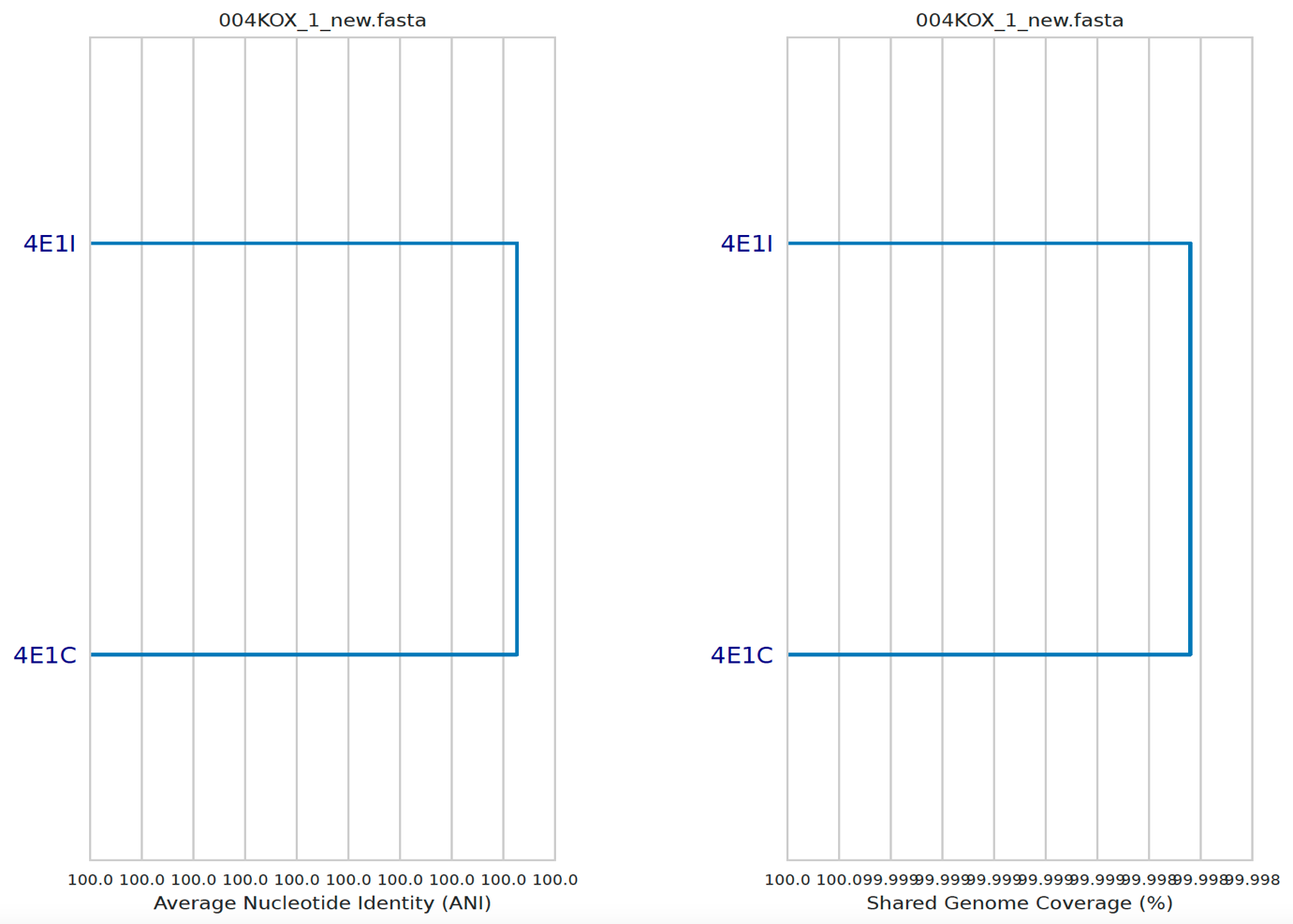
**
